# Cleaner fuels for ships provide public health benefits with climate tradeoffs

**DOI:** 10.1038/s41467-017-02774-9

**Published:** 2018-02-06

**Authors:** Mikhail Sofiev, James J. Winebrake, Lasse Johansson, Edward W. Carr, Marje Prank, Joana Soares, Julius Vira, Rostislav Kouznetsov, Jukka-Pekka Jalkanen, James J. Corbett

**Affiliations:** 1 Atmospheric Composition Research, Finnish Meteorological Institute, P.O. Box 503 FI-00101 Helsinki, Finland; 2 Rochester Institute of Technology, Rochester, NY 14623 USA; 3 Energy and Environmental Research Associates, LLC, Pittsford, NY 14534 USA; 4 University of Delaware, 305 Robinson Hall, Newark, DE 19711 USA

## Abstract

We evaluate public health and climate impacts of low-sulphur fuels in global shipping. Using high-resolution emissions inventories, integrated atmospheric models, and health risk functions, we assess ship-related PM_2.5_ pollution impacts in 2020 with and without the use of low-sulphur fuels. Cleaner marine fuels will reduce ship-related premature mortality and morbidity by 34 and 54%, respectively, representing a ~ 2.6% global reduction in PM_2.5_ cardiovascular and lung cancer deaths and a ~3.6% global reduction in childhood asthma. Despite these reductions, low-sulphur marine fuels will still account for ~250k deaths and ~6.4 M childhood asthma cases annually, and more stringent standards beyond 2020 may provide additional health benefits. Lower sulphur fuels also reduce radiative cooling from ship aerosols by ~80%, equating to a ~3% increase in current estimates of total anthropogenic forcing. Therefore, stronger international shipping policies may need to achieve climate and health targets by jointly reducing greenhouse gases and air pollution.

## Abstract (web summary)

Aerosol pollution from shipping contributes to cooling but also leads to premature mortality and morbidity. Here the authors combine emission inventories, atmospheric models and health risk functions to show how cleaner marine fuels will reduce premature deaths and childhood asthma but results in larger warming.

## Introduction

Ship power systems emit a wide variety of pollutants that have important health and climate change impacts. Fine particulate matter (PM_2.5_), sulphur oxides (SO*x*), and nitrogen oxides (NO*x*) that emerge from ship smokestacks lead to premature mortality and morbidity effects that are well documented^1–10^. In particular, SO*x* emissions form sulphate (SO_4_) aerosols that increase human health risks and contribute to acidification in terrestrial and aquatic environments^11^. Sulphates from ships also participate in regional short-lived aerosol cooling that affects radiative budgets.

Given shipping’s significant contribution to global sulphur inventories (estimated to be 13% of total SO*x* emissions annually^12^), the International Maritime Organization (IMO) proposed new global standards to limit sulphur (S) in fuel oil to 0.5% S (by mass) after January 1, 2020, from the current limit of 3.5% S. These standards will reduce sulphate aerosols and provide health benefits to exposed populations. Sulphate reduction will also directly and indirectly affect atmospheric light scattering and absorption properties, thereby increasing net forcing effects that contribute to climate change^13–15^. These coupled effects raise both environmental and social implications because shipping activity along major trading routes distributes these changes heterogeneously. Thus, policymakers face tradeoffs whereby achieving human health benefits may be associated with climate change consequences^16–18^.

This work presents an integrated global spatial analysis that assesses regional health benefits and net aerosol forcing effects for current and future lower-sulphur marine fuels. Complying with low-sulphur limits for marine fuels reduces ship air pollution and attributable health impacts substantially. Prior to cleaner ship fuels, ship-related health impacts include ~400,000 premature deaths from lung cancer and cardiovascular disease and ~ 14 million childhood asthma cases annually. Reduced PM_2.5_ from marine engine combustion mitigates ship-related premature mortality and morbidity by 34 and 54%, respectively. Reduced aerosol radiative cooling attributable to ship emissions accompanies health benefits from lower-sulphur fuels. Cleaner fuels reduce radiative cooling from ship aerosols by ~80% (71 mW m^−2^) due to lower direct aerosol cooling (−3.9 mW m^−2^) and lower cloud albedo (−67 mW m^−2^). Local intensities of these changes in health and climate directly relate to the major patterns of ship traffic along major trade routes and continental coastlines.

## Results

### Overview of approach

On the basis of 2015 Automatic Identification System (AIS) data on shipping traffic (over 65,000 IMO registered vessels and over 7.6 billion motion records), we project 2020 geospatial shipping emissions inventories with and without implementation of the proposed standards. Using global chemical transport models with very high spatial and temporal resolution (10 × 10 km × 3 h), we evaluate the public health and climate forcing consequences of low-sulphur marine fuel policy implementation. Atmospheric transport and transformations of ship-emitted pollutants serve as inputs to modelling health effects, and to estimating direct and indirect radiative forcing potential. All computations incorporate existing regulations that limit sulphur emissions from ships in designated Sulphur Emissions Control Areas (SECAs). We report results of advanced shipping emissions inventories for 2015 and 2020 scenarios, high-resolution spatial and temporal chemistry-transport port model runs, health outcomes using linear and log-linear concentration–response functions, and radiative transfer evaluations of direct and indirect aerosol forcing changes.

### Global shipping emissions in 2015 with projections to 2020

Global ship emissions were calculated using the Ship Traffic Emissions Assessment Model (STEAM) model^19–21^ and 2015 AIS ship traffic (see Methods). Emissions were projected to 2020 using vessel-type-specific annual growth rates (MEPC 70/5/3^22, 23^ Table 166). Annual totals for emissions from global shipping for 2015 and 2020 are presented in Table 1, comparable with the 2012 results described in the Third IMO GHG Study^12^. Projected business-as-usual (BAU) emission results of the global fleet in 2020 are consistent with those of the Third IMO GHG Study, mainly reflecting growth assumptions; as shown in Table 1, emissions reductions associated with implementation of a low-sulphur fuel standard in 2020 (2020 Action) affect both SO_2_ and PM (via reduction of sulphate emissions).

**Table 1:** Summary of emissions (and fuel consumption) of this work using STEAM for 2020 compared with Third IMO GHG Study (Smith et al.^12^) and all non-shipping emissions

Pollutant (000 tonnes)	Third IMO GHG study estimate for 2012	2015 estimate	2020 BAU without IMO standard	2020 action with IMO standard	2010 non-shipping emissons HTAPV2/MEIC^73^
NO_*X*_	19,000	20,100	21,300	21,300	75,310
SO_*X*_	10,200	11,500	11,000	2500	99,071
PM^a^	1400	1540	1500	770	17,338
CO_2_	938,000	814,000	860,000	870,000	—
Fuel Usage^b^	254,000 (t-d) 300,000 (b-u)	263,000	277,000	274,000	—

^a^ PM estimates in this work include speciated fractions for modelling, including sulphate, elemental carbon, organic carbon, and ash, as appropriate for the source characteristics. For ships, PM includes sulphate formed from gaseous emissions of SO_*X*_. Nearly all ship-emitted PM falls within the PM_2.5_ size range

^b^ The Third IMO GHG Study reported both top-down (t-d) and bottom-up (b-u) estimations; STEAM methods are activity based, bottom-up

Implementation of the 2020 standards results in ~75% reduction in shipping SO*x* emissions globally, along similar spatial distribution of emissions without the new standards (see Supplementary Figure 1). Supplementary Figure 1 also illustrates the presence of an IMO-approved sulphur emissions control area (SECA) designation for the United States (US) and Canada^24^; a Chinese domestic emissions control area (DECA) designation for the Bohai Sea, Yangtze River delta, and Pearl River delta in China^25^; IMO-approved SECA designation in parts of Europe; and additional European Union control requirements under the European Directive^26^. In the European and US/Canadian SECAs, ships are limited to burning 0.1% S fuel within 200 nautical miles of the coast, whereas the limit under the European Directive and in Chinese areas will be 0.5% S from the beginning of 2020 and 2019, respectively. These SECAs will be in force independent of the IMO decision to implement global fuel sulphur standards.

### Impact of low-sulphur standards on particulate matter

**Fig. 1:**
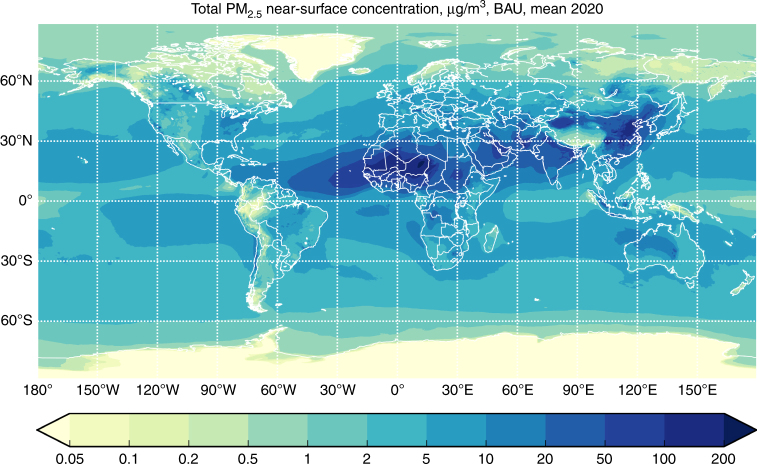
Mean annual PM_2.5_ concentrations from all sources. Model results showing mean annual PM_2.5_ concentrations in micrograms per cubic meter from all sources and with business-as-usual ship emissions in 2020

**Fig. 2:**
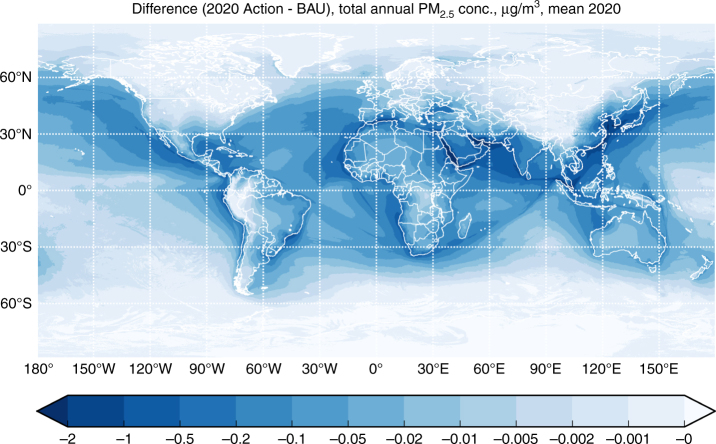
Reduction in annual PM_2.5_ concentrations due to low-sulphur fuel standards. Model results showing the reduction in annual PM_2.5_ concentrations in micrograms per cubic meter due to the implementation of the International Maritime Organization’s global fuel sulphur standard in 2020

Geospatial emissions inventories provide input into the Finnish Meteorological Institute’s (FMI) System for Integrated modeLling of Atmospheric coMposition (SILAM)^27–29^. SILAM was run at geospatial resolution of 10 x 10 km and a temporal resolution of 3 h (see Methods section). We used SILAM to predict pollutant concentrations under the following two scenarios: (1) business-as-usual (BAU), which assumes no implementation of a global, low-sulphur fuel standard; and (2) 2020 Action, which assumes on time implementation of the IMO’s 0.5% S low-sulphur fuel standard. Figure 1 shows the spatial distribution of atmospheric PM_2.5_ near-surface concentrations under the BAU scenario; Fig. 2 presents the difference between the BAU scenario and the 2020 Action scenario. Related concentration maps provided in Supplementary Figure 1 shows consistency with previous distributions at a global level^30^.

Considering all PM sources, typical concentrations of PM_2.5_ amount to a few μg m^−3^, with much higher levels in arid areas and regions with strong fire activity (Fig. 1). Over the ocean, the major component of PM_2.5_ is sea salt (over 50–75%), whereas over land terrestrial emissions are the major contributor. Ship contributions are visible mainly in the open ocean and over the busiest sea-lanes. Stricter limits on sulphur content in ship fuel decrease sulphate concentrations (2–4 μg SO_4_ m^−3^ annual mean), leading to significant reduction of PM_2.5_ in the vicinity of busy ship lanes (Fig. 2). However, total aerosol load reduction is limited since sulphates constitute on average less than 15% of total PM in the air (compare concentrations in Figs. 1 and 2).

Chemical links between sulphate and nitrate species resulted in some ammonia becoming available for forming ammonium nitrate, partly offsetting the sulphate aerosol reductions. However, this effect is small and the offset only exceeds 1% around the east coast of China, with a maximum of 15% over the Yellow Sea.

### Health impacts of global shipping

We apply concentration–response (C–R) functions to 2020 population projections to estimate health impacts due to ship emissions in the BAU case and the 2020 Action case (see Methods section). We calculate adult mortality from lung cancer and cardiovascular disease and childhood asthma morbidity, and report results using a linear C–R function as discussed in Lepeule et al.^31^, and Zheng et al.^31, 32^. The vast majority of PM_2.5_ exposure concentrations in our study area represent conditions similar to those in the Six Cities Study, indicating that functions derived from that study can also apply to our study.

**Fig. 3:**
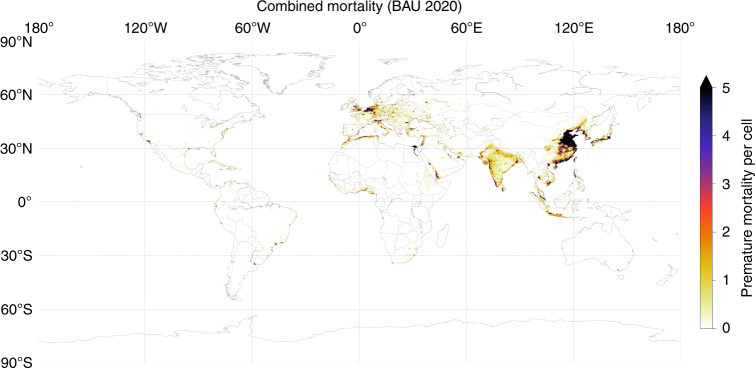
Mortality due to ship emissions under a business-as-usual case. Map of combined mortality (from cardiovascular disease and lung cancer) due to PM_2.5_ emissions from ships under a business-as-usual case for 2020. The in-grid-cell minimum and maximum mortality estimates are 0 and 2550, respectively

**Fig. 4:**
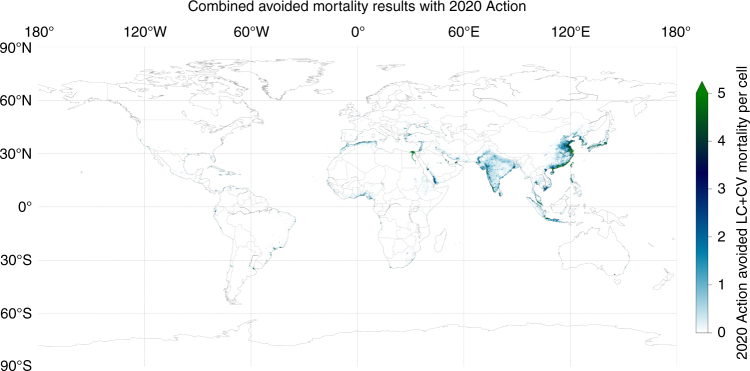
Avoided mortality due to fuel sulphur standards. Map of avoided mortality (cardiovascular disease and lung cancer) from reduced ship PM_2.5_ emissions due to implementation of the International Maritime Organization’s low-sulphur fuel standards in 2020. Annual avoided mortality minimum and maximum are 0 and 800, respectively

Total premature mortality due to shipping in the 2020 BAU case is 403,300 per year (range of 212,300–595,400 based on the 95% confidence interval for relative risk); BAU mortality distribution is shown in Fig. 3. Total avoided premature mortality in 2020 with implementation of the low-sulphur fuel standards is expected to be 266,300 per year (range of 138,500–395,700), a reduction of ~ 34%); avoided mortality distribution is shown in Fig. 4. Childhood asthma morbidity due to shipping declines by 54%, from 14 million children affected in the BAU case, to 6.4 million children in the 2020 Action case (see Supplementary Note 1, Supplementary Figure 2. Detailed results are in Table 2, and Supplementary Table 1 and Supplementary Table 2 report regional results.

**Table 2:** Estimated annual health impacts of global shipping in 2020 in the BAU case and the 2020 Action case with IMO low-sulphur fuel standard, where parentheses indicate 95% confidence intervals based on relative risk calculations

Scenario results (linear C–R model)	Mortality estimate (annul premature adult deaths^a^)		Childhood asthma (million cases)
BAU 2020 (No implementation of global 0.5% S fuel standard)	CV mortality	349,000 (CI 95%: 200,300; 501,800)	14.0 (CI 95%: 7.5; 21.0)
	LC mortality	54,300 (CI 95%: 12,000; 93,600)	
	Combined mortality	403,300 (CI 95%: 212,300; 595,400)	
2020 Action (Implementation of global 0.5% S fuel standard in 2020)	CV mortality	226,800 (CI 95%: 129,800; 327,000)	6.4 (CI 95%: 4.1; 11.5)
	LC mortality	39,500 (CI 95%: 8,700; 68,700)	
	Combined mortality	266,300 (CI 95%: 138,500; 395,700)	
Health benefit of 2020 Action	Avoided mortality^b^		Avoided morbidity
	CV: 122,200 (CI 95%: 70,500; 174,800)		7.6 (CI 95%: 3.4; 9.6)
	LC: 14,800 (CI 95%: 3,300: 24,900)		
	Combined: 137,000 (CI 95%: 73,800; 199,700)		

CV cardiovascular disease, LC lung cancer, CI 95% 95 percent confidence interval

^a^ Values for annual premature mortality are rounded to nearest 100; values for annual childhood asthma morbidity rounded to nearest 100,000

^b^ Differences between avoided health impacts and scenario differences attributed to rounding

More than 97% of the adult mortality benefits from ship emissions reductions will be in Asia (80%), Africa (12%), and Latin America and the Caribbean (5%). More than 98% of the childhood morbidity benefits from ship emissions reductions also occur in Asia (54%), Africa (33%), and Latin America and the Caribbean (12%). The different distributions are primarily due to the different distributions of adult and youth populations among nations. Europe, North America, and Oceania combined will receive <3% and <2% of the mortality and morbidity benefits of the global sulphur standard, respectively. This is primarily due to existing legislation (with or without the global standards) in North America, the Baltic Sea, the North Sea, the English Channel, and EU sea areas in general (see Supplementary Note 2 for regional tables).

Our assessment of the global health burden due to air pollution from ships are much higher than prior assessments primarily due to improved geospatial resolution of global models, updated inputs, and the use of linear C–R functions (see Methods section). First, by applying higher spatial and temporal resolution using the 2020 STEAM inventories and SILAM chemistry transport model, we more precisely assess proximal exposure concentrations, better quantifying peak exposure to at-risk communities (see Supplementary Note 2, Supplementary Figure 3). Second, the updates provided by Lepeule, et al.^31^, increase the health risks attributable to PM compared to previous assessments; e.g., the attributable fraction for lung cancer due to PM_2.5_ exposure increases by a factor of 2.5. Third, we adopt the so-called linear relative risk function by Lepeule, et al.^31^, which differs from the log-linear functions of Pope, et al.^33^ and Ostro^34^ used in our previous health burden studies. The linear formulation produces characteristically higher health burden estimates for the range of ship pollution concentrations modelled (see Methods section and Supplementary Note 1). At higher concentrations, the combination of updated *β* coefficients and C–R functional form can increase the in-cell health burden estimate by orders of magnitude. Supplementary Table 3 presents results using log-linear C–R function. Supplementary Tables 4 and 5 present regional mortality and asthma results using log-linear C–R function, respectively. Lung cancer mortality using the linear function is more than three times the log-linear C–R results in Supplementary Table 3, and more than seven times the lung cancer estimates using coarser resolution inputs in Winebrake et al.^10^. (Note that Winebrake et al.^10^ computed premature cardiopulmonary mortality and cannot be compared directly with cardiovascular mortality as discussed in Methods section).

Our linear function BAU 2020 estimates in the East Asia region are an order of magnitude higher than the health burden estimated for 2013 by Liu et al.^7^ (238,100 vs. 24,000 air pollution deaths from shipping). This is clearly a result of the different choices of C–R functions, where Liu et al. use an assortment of relative risk functions within their exposure–response model based on Burnett et al.^35^. For our 2020 Action case, global controls for sulphur standards would reduce the shipping health burden in the East Asia domain of Liu et al. by 25% (with 34% reduction of all Asia as shown in Supplementary Table 1). Calculations using the log-linear C–R function in Supplementary Note 3 are 45% higher than the health burden estimated by Liu et al., and their estimates fall within the log-linear 95% confidence interval.

Ship pollution matters in the context of total health impacts from ambient (outdoor) air pollution, with or without the new standards. Despite demonstrated health benefits associated with a low-sulphur fuel standard, ship traffic using cleaner fuel will produce air pollution impacting mortality and morbidity in proximal coastal communities. The World Health Organization (WHO) estimates ~3 million deaths in 2012 attributed to ambient air pollution^36^, with 1.48 million attributed to lung cancer and cardiovascular disease; the World Bank estimates 5.5 million deaths in 2013 attributed to both household and ambient air pollution (2.9 million due to ambient PM_2.5_)^37^. Other scientific peer-reviewed journal papers report similar health burdens due to ambient PM, with estimates ranging from 2.2 to 3.3 million deaths annually^38–42^.

In terms of childhood asthma, the results are also quite significant. Under our BAU case and linear risk function, shipping emissions lead to ~14 million childhood asthma cases annually. Further, even with the low-sulphur fuel standards proposed by IMO, ships are still responsible for ~6.4 M childhood asthma cases annually. The 2014 Global Asthma Study estimated “as many as 334 million people in the world have asthma,” and statistics indicate 26% of the world population is 14 years or younger^43^. Without adjusting for higher prevalence for asthma among young and old persons, direct application of population statistics suggest 86 million children could suffer from asthma, and our linear-function results suggest that shipping is currently responsible for ~16% of these cases (~7.5% if the new IMO standards are implemented).

Most synthesis reports reference sources using log-linear C–R functions and matched *β*-coefficients^35–42^, and some meta-analyses do not explicitly report underlying C–R functions informing their health assessments. We can make bounding comparisons of the contribution of ship pollution to global cardiovascular and lung cancer mortality and asthma morbidity assuming that our log-linear specifications compare better with other studies (see Supplementary Note 3). Under the BAU scenario, shipping accounts for about 3.6% of WHO and World Bank mortality estimates from ambient air pollution, and nearly 7% of lung and cardiovascular disease mortality; shipping pollution under the BAU scenario contributes to ~3.6% of childhood asthma morbidity. Under the 2020 Action scenario, shipping would contribute about 2% of WHO and World Bank air pollution total mortality from ambient air pollution, about 4% of the lung cancer and cardiovascular disease mortality, and about 2.5% of childhood asthma. Cleaner ship fuels reduce exposure to ambient PM_2.5_ air pollution, reducing total ambient air pollution deaths from lung cancer and cardiovascular disease by ~2.6% and reducing total childhood asthma by ~3%. Along major shipping lanes and ports near densely populated coastal regions, these health impacts are much greater.

### Radiative forcing due to low-sulphur fuel

Global low-sulphur fuel standards also impose climate forcing consequences. Sulphates from ship emissions reduce radiative flux at the earth’s surface (i.e., contribute a cooling effect), previously estimated in the range of −47 to −8 mW m^−2^ (direct radiative effect) and −600 to −38 mW m^−2^ (indirect radiative effects)^15, 30, 44–46^.

Our analysis covered direct and the first-indirect effects (i.e., impact on the number concentration and size of cloud droplets and, consequently, cloud albedo via the Twomey effect). We estimated these effects for a variety of assumptions regarding the aerosol properties and meteorological conditions. We found that the global average of the direct radiative forcing of ship-emitted sulphates and nitrates strongly depends on the assumption of the single-scattering albedo (SSA) of the particles, consistent with Lohman et al.^47^ and Lesins et al.^47, 48^. The cooling under the BAU scenario is about −5.2 mW m^−2^ and −1.5 mW m^−2^ for sulphate and nitrate scattering aerosols, respectively (Table 3). Comparing BAU and 2020 Action scenarios, reduction of sulphates will lead to over 4 mW m^−2^ of reduced cooling, whereas the nitrate concentrations will increase due to the compensation effect, thereby adding extra cooling of almost 0.2 mW m^−2^. The net reduced direct cooling will be 3.9 mW m^−2^ for scattering particles.

**Table 3:** Summary of change in radiative forcing due to aerosol effects in BAU and 2020 Action scenarios

Radiative forcing (mW m^−2^)	Direct effect	Indirect effect	Net aerosol forcing^a^
Scenario			
BAU	−6.7	−86	−93
2020 action	−2.8	−19	−22
Net change	3.9	67	71
	Percent change in radiative forcing (positive percentage = increased warming)		
Shipping	75%	81%	81%
Transport^b^	2.1%	36%	38%
All human activity^c^	0.2%	3.6%	3.8%

^a^ Net sums may differ from observed sum in table due to rounding

^b^ 15% of anthropogenic radiative forcing, per Fuglesvedt^46^

^c^ Comparative calculation uses ~1.8 W m^−2^ vs. preindustrial level, per IPCC^13^

Estimation of the indirect effects remains very uncertain and depends on the assumption on the distribution of the ship-induced sulphate aerosols. We quantified the first-indirect effect using empirical relationships between the sulphate and sea salt concentrations derived from several cloud chemistry observation campaigns^49–51^ (see Methods section). This allowed estimating the sensitivity of the forcing in terms of the vertical in-cloud distribution of the sulphate aerosols. In order to compute an envelope of the possible variations, we considered two extreme cases: (1) when the sulphates are well mixed with the cloud water, i.e., the in-cloud convection mixes them perfectly; and, (2) when the in-cloud convection does not affect the sulphate vertical profiles. In the latter case, the sulphates and cloud-water vertical profiles are largely detached, and the upper levels of the clouds are practically not polluted by sulphates. For the well-mixed case, the cooling by ship-induced sulphates amounts to −86 mW m^−2^ for our BAU scenario and −19 mW m^−2^ for 2020 Action scenario. This means that the reduced cooling due to these first-indirect effects is 67 mW m^−2^.

Sensitivity simulations for direct and indirect effects highlighted several parameters affecting the final estimates. The direct radiative effect is strongly sensitive to aerosol optical features. The ship-induced sulphates and nitrates in nature are not pure scattering aerosols due to emissions of black carbon as part of PM. Black carbon serves as condensation centres attracting SO_2_ and creating internally mixed particles with black cores coated by sulphates. Sensitivity computations suggested that at SSA of ~0.975 the direct cooling effect of ship plumes is zero, whereas darker aerosols are the warming agents (see Supplementary Figure 5, for results with SSA = 0.999, 0.99, and 0.95). The tipping point at SSA = 0.975 leads to all-zero direct forcing practically everywhere^48^. Therefore, the change of the direct radiative forcing due to low-sulphur standards will strongly depend on the technologies used for reaching the limitations, specifically how control technologies affect black carbon emissions^52, 53^.

Sensitivity of the indirect effect to the vertical profile of in-cloud sulphates seems to be most important to our estimates. In the detached sulphate profiles, the dominant effect of upper cloud layers, which are practically not polluted by sulphates, determines the overall cloud albedo regardless of the pollution at the cloud bottom, resulting in the lower estimate. Therefore, if most of sulphates are located near the cloud bottom, the reduced cooling is much less than for the well-mixed case: 17 mW m^−2^ instead of 69 mW m^−2^ (see Supplementary Note 4, Supplementary Figure 6). With high-resolution simulations, however, the well-mixing assumption is closer to observations, especially in tropical conditions, where horizontal size of the convective clouds is indeed of the order of 10 km. Therefore, the best estimate from this study is 69 mW m^−2^.

The above results are generally consistent with other literature^15, 46, 54, 55^. Thus, Eyring et al.^54^ roughly estimated direct cooling effect of sulphates of −11 to −26 mW m^−2^ in 2030 depending on traffic scenarios^54^, which is well in line with our BAU case plus the increase of ship traffic by 2030. Fuglesvedt et al.^46^ estimated indirect negative forcing from ship sulphate aerosols as −66 mW m^−2^ compare to preindustrial level (uncertainty range (−114, −38) mW m^−2^), which also compares well to our range of the first-indirect effect (−86, −22) mW m^−2^ ^46^. Capaldo et al.^56^ suggested −110 mW m^−2^, but the evaluation against SO_2_ mixing ratio observations showed almost an order of magnitude of over-estimation. A study of Dalsoren et al.^57^ considered a similar set of ship emission scenarios and estimated the total loss of sulphate-induced cooling at −70 mW m^−2^ with 10–15% variability depending on scenario of ship emission in 2030, which is in close agreement with our estimates. However, Dalsoren et al. suggested that direct and indirect effects are similar in amplitude, which differs from our and most other studies, which showed ~10-fold dominance of indirect effect(s).

Our BAU 2020 direct radiative forcing estimates are also consistent with the estimates by Liu et al.^7^ in East Asia^7^. This work estimates the BAU direct forcing in East Asia averages −23 mW m^−2^ (standard deviation of −14 mW m^−2^), with a region peak and minimum forcing of −55 mW m^−2^ and −1.5 mW m^−2^, respectively. We identify two local maxima for sulphate and nitrate radiative forcing (Fig. 3), one in the East Asia domain aligned with Liu et al., and a second region with greater maxima in the South China Sea. Liu et al. evaluated AIS-derived shipping only within their East Asia study domain, which included only a portion of the shipping traffic in the South China Sea. Our global AIS-derived shipping domain extends the major shipping lanes from mainland China to Singapore, and therefore better represents forcing peaks associated with the full extent of regional shipping routes.

A few studies showed much larger effects than the ones above. Tronstad Lund et al.^15^ based on regression between total sulphur emission and radiative forcing suggested the indirect cooling effect as high as −450 mW m^−2^. However, a relative reduction was estimated as 37–40% in direct and indirect aerosol forcing from fuel changes originally presented by Lauer et al.^58^, about half of the results reported in this work^58^. In turn, Lauer et al.^58^ estimated the direct effect at the same −11 mW m^−2^ level as came from our computations but got the indirect effect varying from −190 to −600 mW m^−2^ (about 75% of these attributed to sulphates) depending on ship emission inventory^58^. A similar study of Righi et al.^59^ showed the range from −280 to −400 mW m^−2^ but used higher ship SO_*x*_ emission of 14 Mton per year. Balkansky et al. (2010) reported shipping direct effect of −20 mW m^−2^, and also pointed out the compensation by direct warming effect of road-traffic carbonaceous aerosols.^44^ For global estimates, including CO_2_, the net reduced cooling of this study amounts to nearly 35% of the total radiative forcing from transport^46^ and over 3% of anthropogenic radiative forcing^13^.

Considering the reasons for the differences from some of the previous work, one can notice a generally lower-sulphur lifetime and global mean burden in the current simulations in comparison with other studies. For example, we estimate a 0.6 Mton sulphur burden (2015), vs. 1.5 Mton sulphur burden (mean 1999–2004) assumed by Lauer, et al. Accounting for the lower emission of SO_x_ in 2015 (111 vs. 150 Mton per year in Dentener et al.^60^ used by Lauer et al.), one ends up with the mean atmospheric lifetime of sulphur of about 2.8 days in the current study and 3.6 days in Lauer, et al. Species-specific lifetimes in our runs were 1.5 days for SO_2_ (primary sink is oxidation to SO_4_) and 2.8 days for sulphates, i.e. dry and wet deposition of sulphates are the primary mechanisms controlling the total-sulphur burden in SILAM. These lifetimes are within the range of values reported by other models^61^, with the SILAM estimate being more on the lower end. Evaluation of SILAM is outlined in Supplementary Note 5.

With our results comparing BAU and 2020 Action scenarios, Table 3 presents the estimated change in the radiative forcing from cleaner ship fuels on the following: (a) the shipping sector, (b) the transportation sector, and (c)all anthropogenic activity.

**Fig. 5:**
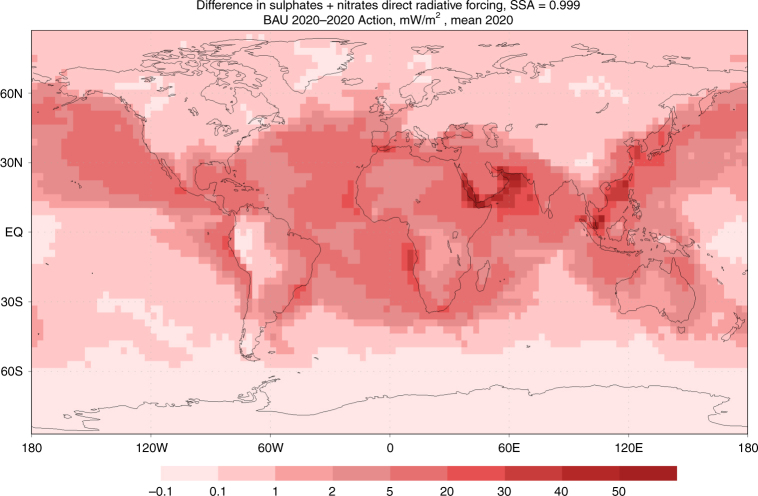
Direct radiative forcing due to low-sulphur fuel standards. Direct radiative forcing in mW m^−^^2^ at the top of the atmosphere from scattering sulphate and nitrate aerosols due to implementation of the International Maritime Organization’s low-sulphur fuel standards for ships. Global mean is 3.9 mW m^−2^

The cleaner fuels will also shift the composition of the forcing aerosols. In the BAU scenario, over two-thirds of the cooling is due to sulphates, whereas the contributions of sulphates and nitrates are practically the same under the 2020 Action scenario. Similar to patterns of near-surface PM_2.5_ concentrations, highly heterogeneous ship traffic density and meteorological conditions determine the radiative forcing patterns (Fig. 5). In particular, the most-significant regional direct radiative effect is over the Persian Gulf, where very dense emissions coincide with large fraction of cloud-free days. Shipping in Southeast Asia is stronger and leads to higher ship-induced PM_2.5_ concentrations covering wider area (Fig. 1). However, the resulting direct radiative forcing is lower than in the Persian Gulf region due to a larger number of cloudy days. For the indirect forcing, the pattern is likely to be the opposite: the effect will be more pronounced in cloudy regions of South-East Asia.

## Discussion

Global compliance with 2020 marine fuel-sulphur standards will reduce annual premature mortality and morbidity in nations across the globe. We demonstrate significant mortality and morbidity benefits from low-sulphur ship fuels in densely populated, major-trading nations. Additionally, even coastal nations, which are not engaged heavily in international trade will benefit, if these nations are proximal to major shipping routes. The distributional impacts associated with air pollution health burdens and the benefits of new standards should be considered in future research. Health burdens and benefits are accompanied by important changes in the regional distribution and global net radiative forcing due to ship-emitted aerosols, which can itself lead to potential health impacts associated with a warmer planet.

Even with cleaner marine fuels after 2020, shipping activity will continue to produce harmful air emissions and greenhouse gases. Notwithstanding the first meaningful global fuel-sulphur controls since fleets converted from coal to petroleum byproducts, energy use in global trade is expected to increase, along with air emissions from shipping^12^. Given that low-sulphur marine fuels still account annually for ~250k deaths (CI 95% ~139k; ~396k) and ~6.4 M childhood asthma cases (CI 95% ~4.1 to 11.5 million), additional reductions beyond 2020 standards may prove beneficial.

Sulphate-specific impacts quantified here cannot be posed strictly as offsetting tradeoffs. Emission reductions of sulphur aerosols by ships using cleaner marine fuels may offer collateral health and climate benefits that merit quantification beyond this work. For example, 2020 compliant fuels may enable or be accompanied with other PM_2.5_ emission reductions, such as organic carbon particles and black carbon particles which contribute to strongly warming effects^62^. Moreover, many control technologies for harmful particulates and ozone precursor emissions perform better under low-sulphur combustion conditions. In a broader context, near-term air-quality health implications and longer-term health implications related to climate forcing deserve joint consideration.

Ship emissions impacts reported here will inform evaluation of additional air pollutant health and climate impacts from shipping activities, and future shipping policies to achieve environmental stewardship goals in shipping and related transportation sectors serving a global economy. International policymaking efforts jointly pursuing air pollution health benefits and climate targets may increase the urgency for continued progress to control and reduce greenhouse gases.

## Methods

### Geospatial emissions inventories

For this research, we constructed geospatial shipping emissions inventories under the following two scenarios: (1) a business-as-usual (BAU) scenario, where we assume shipping emissions are not constrained by a global fuel sulphur standard; and (2) a policy implementation case (2020 Action), where the IMO MARPOL VI fuel sulphur standard goes into effect in 2020. The emissions inventory was facilitated by the use of the Ship Traffic Emission Assessment Model (STEAM), which has been used in similar types of work^6, 12, 19–21, 63–65^. The STEAM model combines 2015 AIS data on shipping routes and volume as well as vessel technical data from IHS Fairplay, with peer-reviewed energy use and emissions equations to construct a geospatial emissions inventory for global shipping. Propulsion energy is evaluated with the Hollenbach resistance calculation method, which is based on tank tests^66^. The STEAM model employs a range of emissions factors, specific to fuel types, engine types, and engine load, and Table 4 presents a range of these values with notes below the table.

**Table 4:** Emission factors used for this study^a^

**Emission**	**Emission factor (g** **kW**^**−1**^ **h), Normal (80%) load**	**Emission factor (g ** **kW** ^**−1**^ ** h), low (25%) load**
NO_*x*_^b^		
Tier 1 engine	17 (SSD), 12.9 (MSD)^b^, 9.8 (HSD)	17 (SSD), 12.9 (MSD)^b^, 9.8 (HSD)
Tier 2	14.4 (SSD), 10.5 (MSD), 7.7 (HSD)	14.4 (SSD), 10.5 (MSD), 7.7 (HSD)
Tier 3	3.4 (SSD), 2.6 (MSD), 2 (HSD)	3.4 (SSD), 2.6 (MSD), 2 (HSD)
SO_*x*_^c^		
0.1% sulphur (S)	0.48 (MDO/MGO: SFOC 250 g kW^−1^ h)	0.54 (MDO/MGO SFOC 282 g kW^−1^ h)
0.5% S	2.40 (MDO/MGO: SFOC 250 g kW^−1^ h)	2.7 (MDO/MGO SFOC 282 g kW^−1^ h)
2.7% S	8.35 (HFO: SFOC 165 g kW^−1^ h)	9.42 (HFO: SFOC 186 g kW^−1^ h)
CO	0.54	2.18
PM		
0.1% S	0.38	0.43
0.5% S	0.50	0.57
2.7% S	1.19	1.35
CO_2_^d^		
HFO	515 (SFOC 165 g kW^−1^ h)	580 (SFOC 186 g kW^−1^ h)
MDO/MGO	803 (SFOC 250 g kW^−1^ h)	905 (SFOC 282 g kW^−1^ h)

SSD slow speed diesel, MSD medium speed diesel, HSD high speed diesel, MDO marine distillate oil, MGO marine gas oil, SFOC specific fuel oil consumption, HFO heavy (residual) fuel oil

^a^ Values only indicate the range of values applied on case-by-case basis because fuel consumption and emissions depend on engine load and specific fuel oil consumption (SFOC), calculated from vessel-specific automated identification system (AIS) data, as described in published literature for STEAM^20, 21, 63, 64^

^b^ As defined in MARPOL Annex VI, Regulation 13. For MSD, crankshaft rpm of 514 is assumed in this example, but engine specific values are used in each case. For Tier 0 engines, 110% of Tier I value is used

^c^ Part of sulphur is as gaseous SO_2_ and part is in aerosol SO_4_. The emission factors listed for SO_*x*_ contain the gaseous emission part, the aerosol sulphur has been subtracted to maintain mass balance of sulphur

^d^ SFOC changes as a function of engine load.0 The values listed include this effect and includes the differences in carbon content between HFO and MDO/MGO

Emission factors for NO_*x*_ depend on engine crankshaft speed (rpm) and age. The IMO Tiers are applied for engines, where Tier 0 follows the definitions of the Third IMO GHG study^12^, and where Tiers 1–3 follow the functions defined in MARPOL VI.

Emission factors for sulphur oxides (SO_*x*_) are determined from fuel sulphur content (% by weight) and the amount of fuel consumed at specific engine load. Part of fuel sulphur is emitted as primary PM and the sulphur fraction included in SO_4_ is subtracted from sulphur available for SO_x_ formation in the atmosphere. All gaseous emissions of sulphur are calculated as SO_2_. Sulphur content for residual fuel use outside of ECA regions in the BAU scenario were assigned 2.7% S, similar to the Third IMO GHG Study; in the 2020 Action scenario, sulphur content was adjusted to 0.5% S. Where lower fuel limits will be in place in 2020 due to current legislation, the maximum sulphur content was set accordingly: (i) the IMO ECA regions (0.1% S)^5^; (ii) the regions covered by the European Directive (0.5% S)^26^; (iii) China legislation applied to the Pearl River Delta, Yangtze River Delta, and Bohai Sea (0.5% S)^25^.

Particulate matter is modelled as dry PM without the associated water, which normally accompanies the sulphate aerosol. The mass of associated water depends on the ambient conditions (temperature, humidity) and the consecutive chemical transport modelling step takes the hygroscopicity of PM into account.

The emission factor for CO_2_ depends on fuel type and specific fuel oil consumption (SFOC) at a specific load point. The base SFOC (at 80% engine load) depends on engine age, power output and stroke type, as defined in the Second IMO GHG Study 2009^67^. Table 4 lists the range of values for HFO and MDO/MGO at high and low engine loads. These examples represent the extremes used in the model for diesel engines. This approach necessitates SFOC modelling as a function of engine load and further details can be found in Jalkanen et al.^20^ and the Third IMO GHG Study^12^.

### Base year inventory adjustments for 2020

Ship inventories for future years were adjusted for the year 2020 using vessel type compound annual growth rates (Table 5), which are consistent with energy use base case growth rates used in the 2016 IMO FAS (see MEPC 70/5/6^23^, Table 166). These growth rates produce future year inventories for ship energy demand and emissions that are lower than some other demand estimates submitted to MEPC-70 by observer delegations [MEPC 70/5/5]^68^; if higher energy demand estimates were used, the health impact from uncontrolled sulphur levels would be greater. The projected emission results of the global fleet in 2020 are similar to those of the Third IMO GHG Study. Largest differences are because of reduction of sulphur in marine fuels, which has an impact on SO_*x*_ and PM emissions.

**Table 5:** Energy-based growth rates derived from Table 166 of 2016 IMO FAS

**Ship type**	**Growth rate**
Dry bulk	1.74%
Liquid bulk	−1.90%
Unitised	2.79%
Passenger	−0.55%
Miscellaneous	0.00%
Total energy	0.95%

### Atmospheric simulations and radiative forcing

The atmospheric simulations have been performed by the chemical transport model SILAM (System for Integrated modeLing of Atmospheric coMposition, http://silam.fmi.fi)^69–72^ with spatial resolution of 0.1 degree longitude-latitude, 13 uneven stacked layers, terrain-following near surface and reaching ~330 hPa at the highest model altitude (see Supplementary Note 5). The output time step was 3 h for all produced quantities: concentrations; dry and wet deposition; and optical thickness at 380 nm, 550 nm, and 1020 nm wavelength.

The non-shipping gridded emissions inventories prepared as input into SILAM include a number of different sources. Anthropogenic source inventories are obtained through HTAPv2+MEIC (Multi-resolution Emission Inventory for China)+REAS (Regional Emission Inventory in Asia)^73^; fires inventories are from IS4FIRES (from FMI, http://is4fires.fmi.fi/72; sea salt inventories are embedded in SILAM^72^; desert dust inventories are embedded in SILAM following the saltation mechanism that Marticorena & Bergametti (1995) advanced in several follow-up works^74–77^; DMS from sea surface is embedded in SILAM after Kettle et al. (2000)^1^; biogenic VOC inventories are from MEGAN-MACC^78^; and aircraft emissions are from RETRO (http://retro-archive.iek.fz-juelich.de/data/documents/reports/D1-6_final.pdf).

Simulations were driven with the meteorological fields of the Integrated Forecasting System (IFS) of the ECMWF with spatial resolution of 0.1 × 0.1 degree and temporal resolution of 3 h. Chemical transformations were computed following the set of gas-phase reactions of CBM-IV carbon-bond mechanism^2^ with updated reaction rates following the IUPAC guidelines (http://iupac.pole-ether.fr/). They were complemented with inorganic aerosol formation reactions described in Sofiev (2000)^79^ for sulphates, nitrates and ammonium. Formation of sulphates was considered as an irreversible reaction, whereas ammonium nitrate was in equilibrium with nitric acid and ammonia. This mechanism was extended with the production of coarse nitrates due to replacement of chlorine on sea salt particles. The organic aerosol precursors were limited to biogenic isoprene and monoterpenes, with emissions computed by Model of Emissions of Gases and Aerosols from Nature (MEGAN). A volatility-basis set (VBS) scheme^80, 81^ was applied to organic aerosol oxidation. All secondary aerosols were considered to be submicron in size under dry conditions but growing if relative humidity goes above species-specific deliquescence points. External mixing of particles was assumed.

Primary particles were represented by anthropogenic elemental and organic carbon as well as mineral PM, all in two size sections: below and above 2.5 μm in diameter. The non-anthropogenic primary aerosols were sea salt (5 bins from 0.01 up to 30 μm), desert dust (4 bins from 0.1 up to 30 μm) and smoke from vegetation fires (3 bins from 0.1 up to 6 μm). Supplementary Figure 1, Supplementary Figure 7, and Supplementary Figure 8 provide further information.

We calculated changes in direct radiative forcing from the change in the aerosol optical depth (AOD) of the atmosphere due to sulphates and nitrates from ships under the BAU and 2020 Action scenarios. We assumed no emission reductions for primary particulates (ash and elemental and organic carbon) and NO_*x*_ under the low-sulphur scenario. Direct radiative forcing was computed as the difference of the upward radiative flux at the top of the atmosphere.

The radiative transfer modelling was completed offline with the Library for radiative transfer (libRadtran) software package for radiative transfer calculations^82^ following the approach of Soares et al.^83^. The libRadTran tool calculates radiances, irradiances, and actinic fluxes for the given optical properties. The Earth radiative balance results from the difference between the incoming and outgoing radiative fluxes at the top of the atmosphere (TOA). The impact of aerosols is assessed by the difference in the upward fluxes at the TOA for the atmosphere with and without particles, for both BAU and 2020 Action scenarios. The calculations were defined with wavelength ranging from 0.2 to 4.0 µm. To limit the amount of calculations, the SILAM AOD fields were averaged to a 3° × 3° grid. All the runs used 3D cloud liquid- and ice-water content, cloud cover and surface albedo taken from the IFS. The aerosol optical depth diagnosed by SILAM was superimposed to the default vertical distribution of aerosols defined in libRadTran. The prescribed aerosol optical properties, such as single scattering albedo, asymmetry factor and Ångström exponent were used. To get integrated radiative fluxes, 3-hourly global fields of outgoing radiation at the TOA were averaged over the whole year.

The first indirect aerosol effect—via impact on the size and the number of cloud droplets and, consequently, on cloud albedo—was computed using the empirical relationship suggested in a series of works^49–51^ and used in modern numerical weather prediction systems such as IFS of ECMWF. The most-uncertain part of it is the multi-component relation, which would include effects of non-sea-salt sulphates combined with the sea salt and non-sulphate anthropogenic aerosols. The approximation formulas in these works suggest a power-law type of dependence, thus claiming zero number concentration of droplets in absence of anthropogenic sulphates. In addition, part of the data with heavy sea salt load was removed from the analysis. A review of the data of Borys et al.^49^ leads to another approximation that describes the whole data set without any withdrawal. It also manifests a physically meaningful asymptote for pristine areas:

$$D_n = a + bC_{{\mathrm{NSS}}} + cC_{{\mathrm{SSLT}}}.$$

Here *D*_*n*_, *C*_NSS_, and *C*_SSLT_ are concentrations of cloud droplets, non-sea-salt sulphates and sea salt, respectively. Fitting constants *a* = 31 # m^−3^, *b* = 93.5 # μg^−1^, *c* = 16.5 # μg^−1^ (where # refers to number of particles).

### Health impact analysis and uncertainty

The process of calculating the health impact analysis follows the general approach discussed in previous work^4, 10^. Prior work applied a mortality risk analysis discussed in Ostro (2004)^34^, which built on work developed out of the United States Harvard Six Cities study conducted earlier by Pope, et al.^33, 84, 85^. The vast majority of PM_2.5_ exposure concentrations in our study area study area represent conditions similar to those in the Harvard Six Cities study, indicating that premature mortality functions derived from that study can also apply to our study (see Supplementary Figure 9 and Supplementary Figure 10). We conduct a similar assessment (updated with new population data, incidence data, and concentration data) using a recently preferred concentration–response (C–R) function from Lepeule, et al.^31^, which updates epidemiology from the Harvard Six Cities study^31^. Recognising that Marshall et al.^86^ specifically notes that the proper functional form is as yet unclear, we present the log-linear C–R function results in Supplementary Note 3 (see global results in Supplementary Table 3, and regional results in Supplementary Table 4 and Supplementary Table 5). The linear C–R function reflects new understanding about the relationship between health impacts and exposure to increased air pollution concentrations and better describes the global range of exposures to global shipping pollution across a long-tailed distribution of PM_2.5_ concentrations (see Supplementary Figure 9). Therefore, the linear C–R function (shown below) provides better estimates of health impacts where i) ambient concentrations of PM_2.5_ are high (e.g., >20 µg m^−3^, WHO guidelines), and ii) major ship traffic contributes more to ambient concentrations (e.g., >10 µg m^−3^). Supplementary Note 3 provides an alternate, log-linear C–R function and *β* coefficients, following Barnett et al. Supplementary Figure 4 illustrates that the log-linear function produces higher relative risk under low initial concentration (*C*_0_) conditions, but appears to imply a saturation effect at higher ambient air pollution concentrations that is not evident in air health impact studies. The log-linear C–R construct enables matched comparison with most of the global health assessments, which do not adopt the linear C–R and *β* recommended by Lepeule’s updates. We focus on cardiovascular and lung cancer mortality responses as identified by Lepeule, et al.^31^. We also extend the health analysis to include for the first time an assessment of childhood asthma morbidity, which uses similar concentration–response equations based on reported asthma incident rates by nation^32^.

Concentration–response functions for chronic exposure adult mortality correspond to impacts for a population cohort aged 30 years or more using Lepeule, et al.^31^. Concentration–response functions for childhood asthma morbidity correspond to impacts for a population cohort aged 14 years or younger using Zheng (2015). Our gridded population data are from NASA’s Socioeconomic Data and Applications Center (SEDAC) Population of the World, Version 4^87^. We apply age cohort fractions directly to the population counts for each nation from the United Nations to determine the age cohort populations by country^88^. The Median Variant values for the year 2020 were used to calculate the 30–99 fraction, based on summing population across all age cohorts at 30 years or older and dividing by the total population.

We calculated country-specific incidence rates for cardiovascular disease and lung cancer using data from the World Health Organization’s Global Health Observatory (GHO), which publishes mortality by age cohort and country, and GLOBALCAN, respectively^89, 90^. In particular, we divided reported deaths from WHO by WHO population estimates for age cohorts 30–99 to calculate an incidence rate for each disease per 100,000 people over 30 years of age. We make a simplifying assumption to attribute all cardiovascular disease and lung cancer deaths to the over 30 population, which is demonstrated to be the case after reviewing cause of death data by age cohort. In cases, where country data were not available through this data source, we used an additional WHO data source that publishes cardiovascular diseases mortality using an age-standardised death rate (ASR) per 100,000. These data were converted to crude (non-ASR) values using a non-ASR:ASR ratio. For lung cancer mortality incidence rates, we use data from GLOBOCAN 2012^89^. To determine overall health impacts associated with ship emissions and the potential effects of IMO policy delays, we calculate expected mortality under the BAU and 2020 Action scenarios by comparing concentration results of these scenarios with a no-shipping case, whereby emissions from ships are removed from the geospatial inventory prior to SILAM processing.

We obtained country-specific incidence rates for childhood asthma using data from the 2014 Global Asthma Report^43^. Specifically, we used the current wheeze data from the ISAAC world map data as reported in the 2014 Global Asthma Report. Zheng et al.^32^ provide relative risk (RR) factors for childhood asthma from exposure to PM2.5 pollution (Table 2 of Zheng), which we convert to *β* coefficients.

We calculated expected mortality and morbidity due to changes in total particulate matter concentrations using approaches mentioned above, consistent with other recent work in this area^7, 10^. The total effect (E) of changes for each grid cell is given as:

$$E = {\mathrm{AF}} \cdot B \cdot P,$$

where *B* represents the incidence rate of the given health effect; *P* is the relevant exposed population; and AF is the attributable fraction of mortality or morbidity to the shipping-related PM pollution, and is given by:

$${\mathrm{AF}} = \frac{\rm RR - 1}{\rm RR}.$$

For a linear C–R model, the response RR is given by the function^31^:

$${\mathrm{RR}} = {\rm e}^{\beta \cdot \left( {C_1 - C_0} \right)}$$

And therefore,

$${\mathrm{AF}} = 1 - {\rm e}^{\beta \cdot \left( {C_0 - C_1} \right)},$$

which leads to

$$E = \left[ {1 - {\rm e}^{\beta \cdot \left( {C_0 - C_1} \right)}} \right] \cdot B \cdot P,$$

where *β* = 0.023111 (95% CI = 0.013103, 0.033647) for cardiovascular mortality; *β = *0.031481 (95% CI = 0.006766, 0.055962) for lung cancer related mortality^31, 35, 85^; and where *β* = 0.002469 (95% CI = 0.001291, 0.003633) for childhood asthma morbidity^32^.

We apply mortality C–R functions in a way that is consistent with previous work published by Winebrake, et al.^10^. There are several important differences in this health analysis compared with previous work; however, similar types of uncertainties affect our health results (some reducing and some increasing uncertainty ranges).

One important uncertainty involves global ship emissions and inventories. Inventory uncertainties using the AIS data and bottom-up models are both reduced and more transparent than previous work that used AMVER and ICOADS data in top-down geospatial inventories, similar to rigorous bottom-up inventory uncertainty analyses reported by the IMO, which also employed an approach similar to the STEAM model^12^. STEAM also applies a fully dynamic spatial-temporal variation of global ship emissions that reduces uncertainty further. Conducting our analysis at a 10 × 10 km resolution reduces spatial uncertainty that existed in previous work. This analysis uses a geospatial resolution considerably more resolved that other comparable work in the literature. To understand how the geospatial resolution affects results, we compare our 10 × 10 km gridded results with results from an analysis done at a 50 × 50 km resolution (Supplementary Figure 7). This comparison demonstrates the heterogeneous regional effects that can result due to geospatial resolution, with notable under-estimation of emissions over large parts of Southeast Asia and Western Europe when using more coarse model outputs.

Another uncertainty is in the specification of a linear C–R function results in higher health impact estimates where shipping pollution concentrations are higher. Based on Lepeule et al.^31^, we use a linear C–R function. Comparison of linear and log-linear C–R functions using different concentration differences demonstrate that the linear model in-grid-cell estimates can be more than an order of magnitude greater than health impacts estimated for the same concentration differences using the log-linear model. Direct comparison of Lepeule and Ostro lung cancer beta-coefficients indicates Lepeule coefficients produce higher estimated mortality impacts. Ostro (2004) estimates the relative risk for lung cancer due to a 1 µg m^−3^ concentration increase (using the so called linear formulation) to be 1.012751, corresponding to an attributable fraction (AF) of 0.01259, and beta coefficient of 0.01267. Lepeule (2012) estimates lung cancer relative risk to be 1.031982, corresponding to an AF of 0.030991 and beta coefficient of 0.031481 Ostro’s 2004 best or central estimate for lung cancer beta-coefficients falls within Lepeule’s 95th percentile confidence range. In addition to lung cancer mortality, this work evaluates cardiovascular mortality, where our prior mortality work evaluated cardiopulmonary mortality. Cardiovascular disease (CVD) is a broad term for a range of diseases affecting the heart and blood vessels that include cardiopulmonary conditions (cardiovascular conditions involving the lungs). Therefore, we observe as expected that Lepeule’s beta coefficients for CVD are not directly comparable with Ostro’s beta coefficients for cardiopulmonary disease, but produce higher estimated mortality impacts.

Lepeule uses additional years’ data to evaluate a broader uncertainty range than Ostro et al., providing a quantitative and consistent update to earlier assessments of regional and global health burden from shipping. Note that Lepeule et al.^31^ not only has a higher value for the coefficient mid-point, but also a wider 95% confidence interval for the beta coefficient compared to previous work.

Employment of best-available incidence rates for each country in a common set of C–R functions applied to the global population assumes pollution response similarity and recognises uncertainty where in-country incidence rates are poorly known or under reported^91, 92^. Where unavailable, global averages incidence rates are used. This source of uncertainty appears to affect asthma morbidity estimates more so than mortality estimates. We observe, for example, that nations with more advanced healthcare systems (i.e., typically wealthier nations) report higher underlying incidence rates for childhood asthma. For mortality, the uncertainty in underlying incidence rates is somewhat mitigated based on results of epidemiological studies that show similar concentration–response patterns across different countries^34, 93, 94^.

A final area of uncertainty includes assumptions about the geographic distribution of populations geospatially. We distribute uniformly across each country the fraction of population under the age of 14 and between the ages of 30 and 99. This may not account for in-country spatial differences in age cohort distributions (e.g., age distributions in coastal communities that may differ from national statistics). Also, this use Knowing that “the burden of asthma, measured by disability and premature death, is greatest in children approaching adolescence (ages 10–14) and the elderly (ages 75–79)”^43^, assigning age cohorts uniformly may produce conservatively low estimates low for childhood asthma morbidity.

### Code availability

The STEAM model is intellectual property of the Finnish Meteorological Institute (FMI) and is not publicly available. This code uses a real-time connection to IHS Fairplay ship register, requiring an annual subscription. Bilateral contracts between data providers and FMI govern access to input data sets that are required for the modelling, such as vessel activity and ship technical data. The work described in this manuscript did not contain modifications of the STEAM model; model description can be found in the references. The SILAM model is an open-code system and is free for research applications. The model can be obtained by contacting persons responsible for the SILAM model development (visit silam.fmi.fi for more information). The health risk functions are fully described in the manuscript and do not require code to replicate; data obtained for this work are also publicly available and cited.

### Data availability

FMI may share STEAM outputs upon request as long as the data are in aggregated form, individual vessels cannot be identified, and the commercial input data cannot be reverse engineered from model outputs. The gridded ship emission files used in this work are available from FMI ftp site upon request to authors. The gridded population data used in this work were taken from NASA’s Socioeconomic Data and Applications Center (SEDAC) Population of the World, Version 4. This data set is available at http://sedac.ciesin.columbia.edu/data/collection/gpw-v4. The Population by Age Group data set can be downloaded at https://esa.un.org/unpd/wpp/Download/Standard/Population/. The cardiovascular disease mortality data using an age-standardised death rate published by WHO were downloaded from http://www.who.int/healthinfo/global_burden_disease/estimates/en/index1.html. Asthma data reporting incidence rates are provided by The Global Asthma Report 2014 at http://globalasthmareport.org/.

## Electronic supplementary material

Supplementary Information
