# Supplementary material for: Cleaner fuels for ships provide public health benefits with climate tradeoffs
Source: Nat Commun. 2018 Feb 6;9:406. doi: 10.1038/s41467-017-02774-9 (PMC5802819; doi:10.1038/s41467-017-02774-9)
Supplement: Supplementary file 1 — Supplementary Information [file 41467_2017_2774_MOESM1_ESM.pdf]

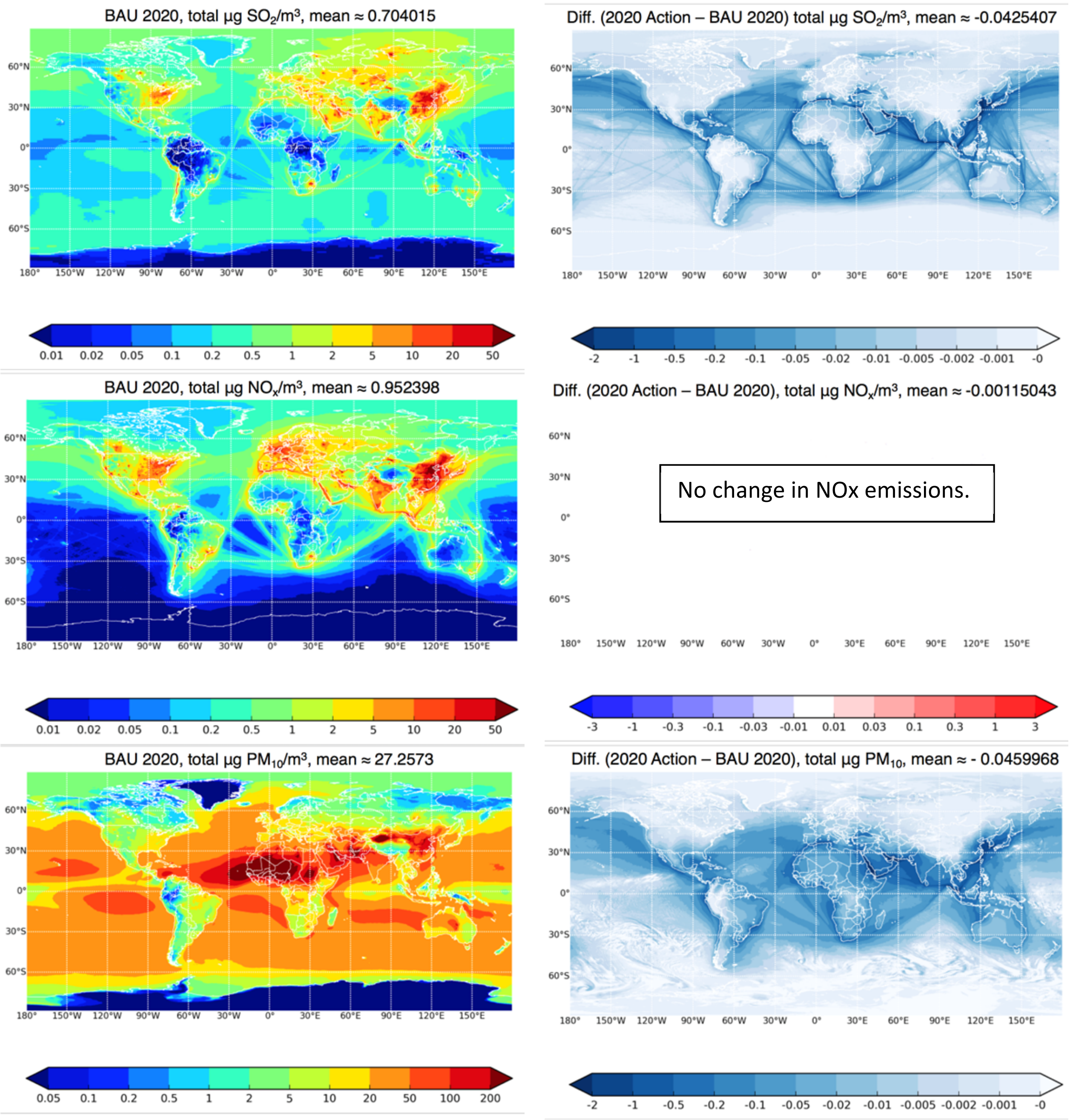

Supplementary Figure 1 : Near-surface concentration maps

Near-surface concentrations (left-hand column) and difference between BAU and ONTIME scenarios (right-hand column) for SO_2, NO_2, PM_{10}.

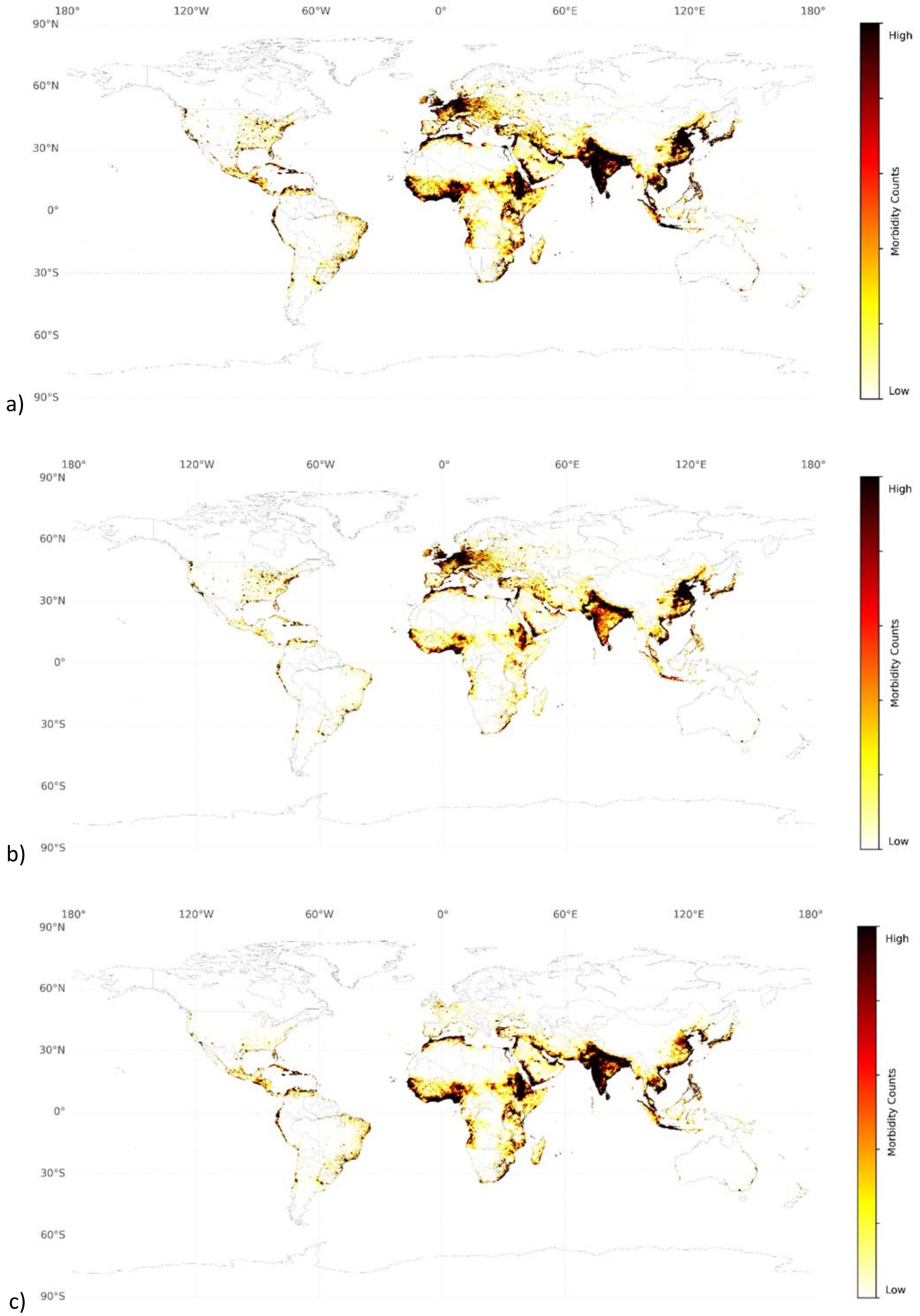

Supplementary Figure 2 : Childhood asthma morbidity maps

Ship-related Childhood Asthma Morbidity Results for a) BAU Case; b) 2020 Action Case; and c) Reduction in Childhood Asthma.

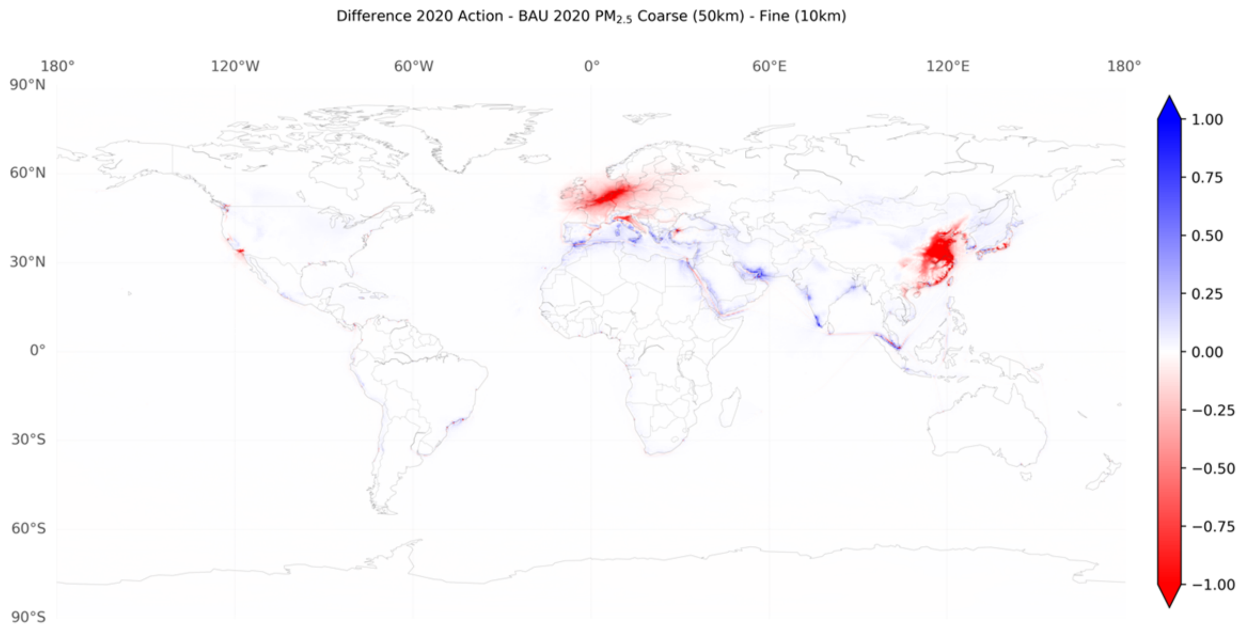

Supplementary Figure 3 : Model resolution comparison map

Comparison of $PM_{2.5}$ concentrations of a highly resolved (10x10km) spatial analysis versus a less resolved case (50x50 km) analysis; negative values (red) demonstrate areas where the 50x50 case results in lower concentrations and positive values (blue) demonstrate areas where the 50x50 case results in higher concentrations.

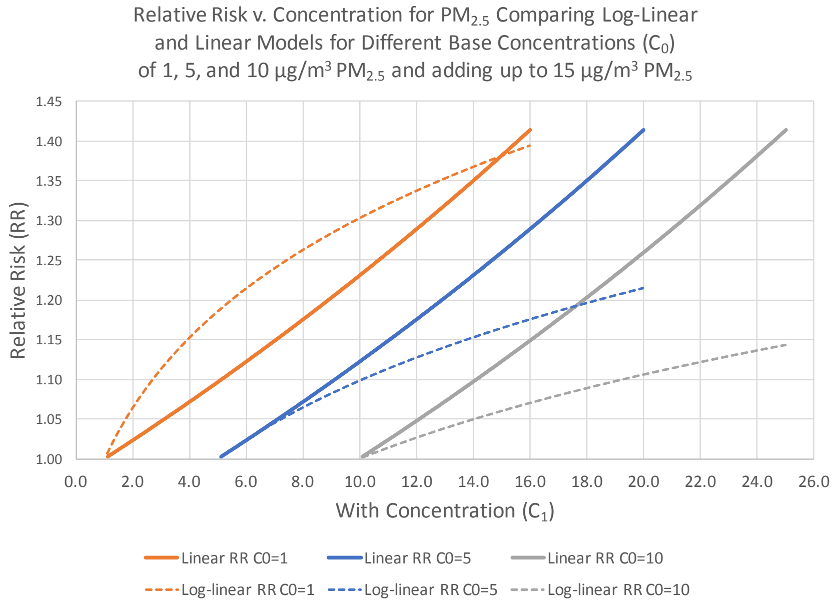

Supplementary Figure 4 : Linear and log-linear risk graph

Comparison of relative risk under linear and log-linear model specifications, with starting concentrations of 1, 5, and 10 µg m^{-3}.

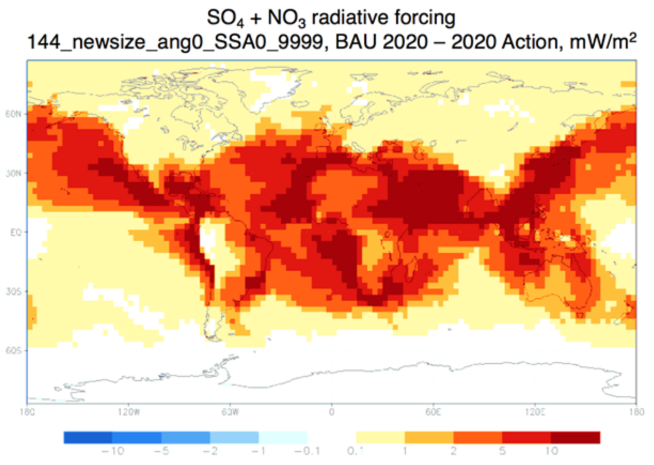

SSA = 0.9999

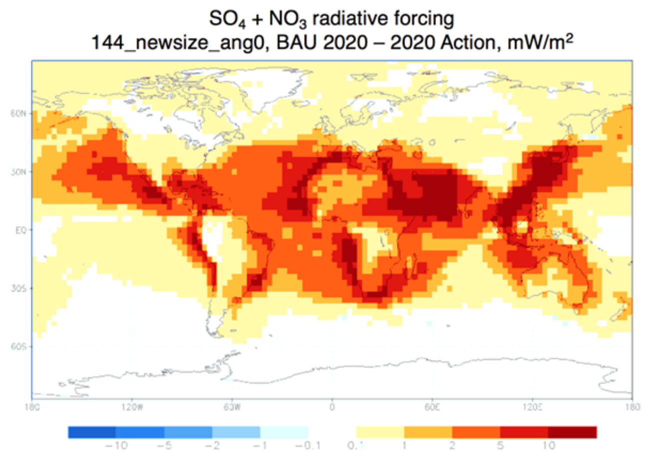

SSA = 0.99

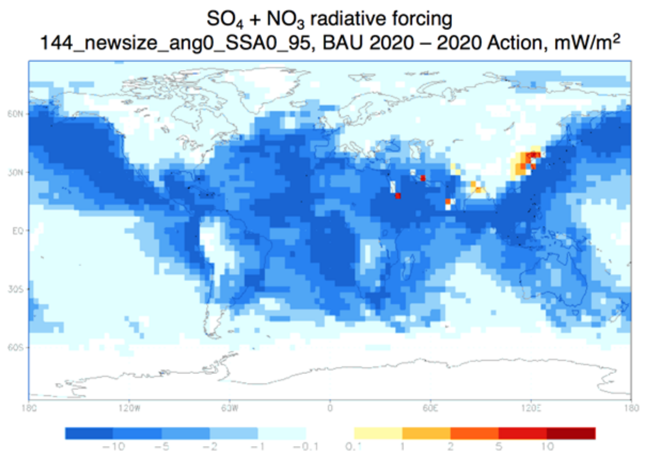

SSA = 0.95

Supplementary Figure 5 : Direct forcing sensitivity

Sensitivity of direct radiative forcing of ship-induced sulphates and nitrates to the particle single-scattering albedo (SSA).

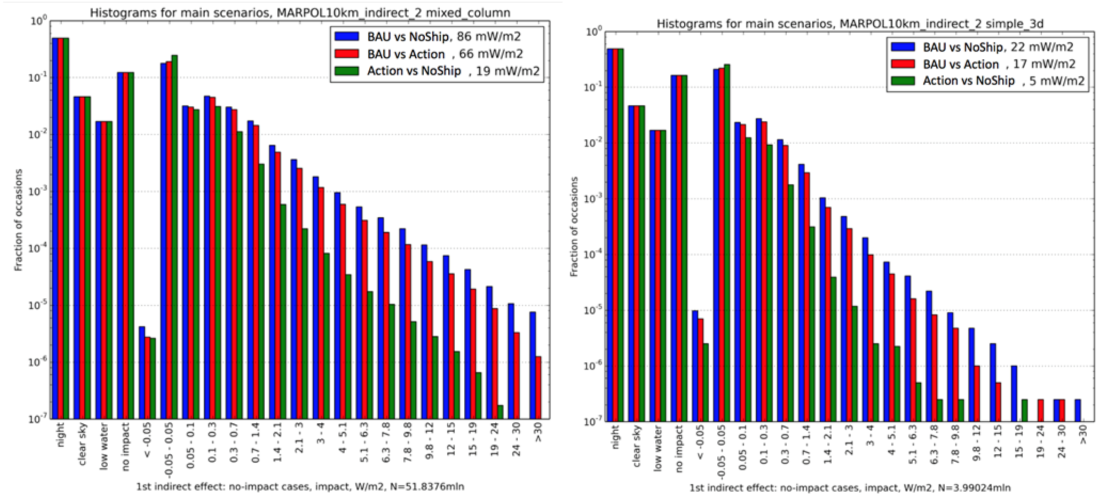

Supplementary Figure 6 : Histograms for indirect forcing

Frequency distribution of the 3-hourly-mean indirect radiative forcing over the globe. Bars from left to right: no-impact cases (night-time, no clouds, very thin clouds, areas not affected by ships), accidental reverse impact due to numerical errors, impact below 50mWm^{-2}, other columns represent the frequency of impact cases up to 30 Wm^{-2}. Left panel: with in-cloud convection, right panel – without it.

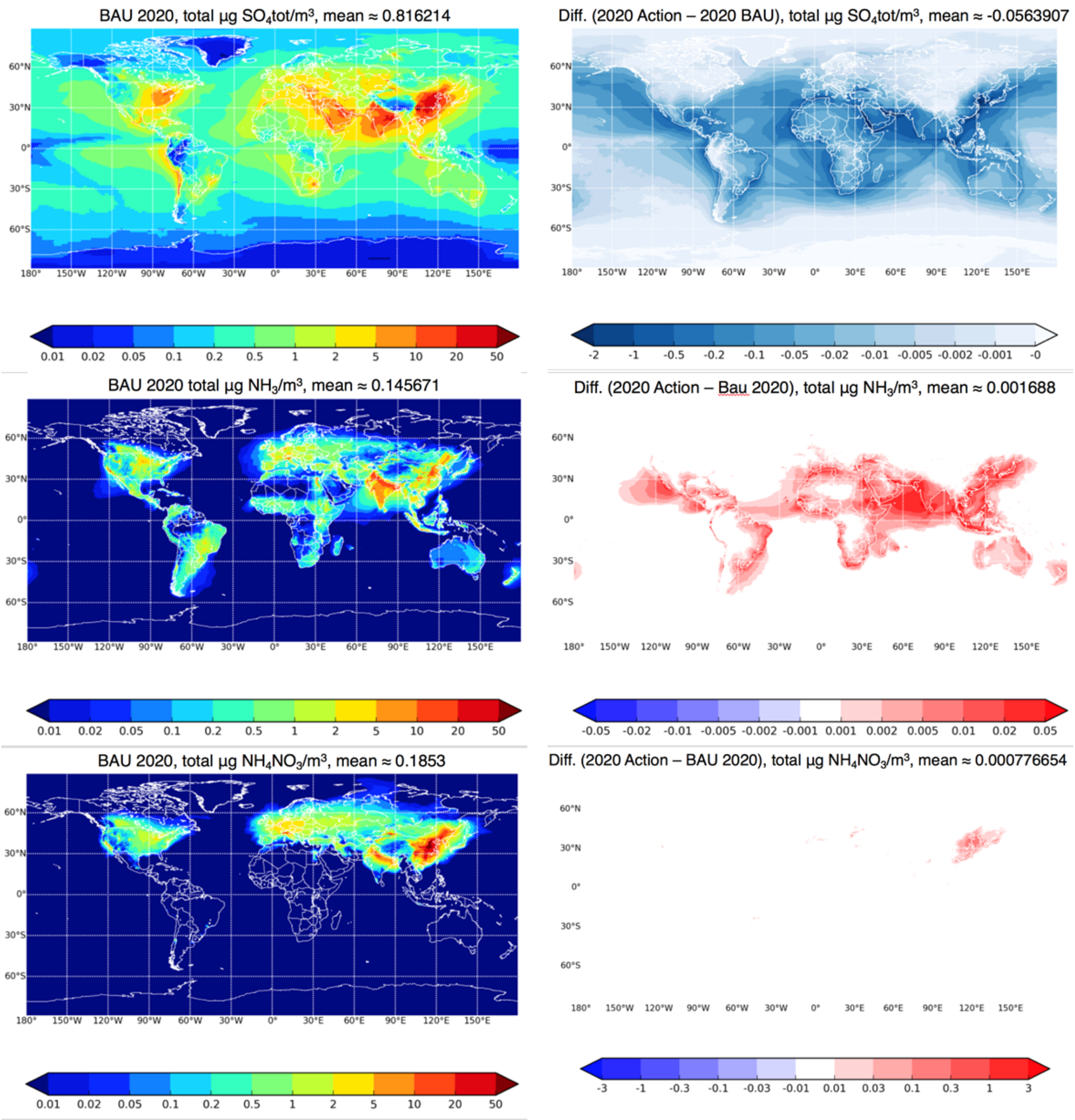

Supplementary Figure 7 : Nitrate-sulphate compensation

Near-surface concentrations (left-hand column) and difference between business-as-usual (BAU) and 2020 Action scenarios (right-hand column) for SO_4, NH_3, NH_4NO_3.

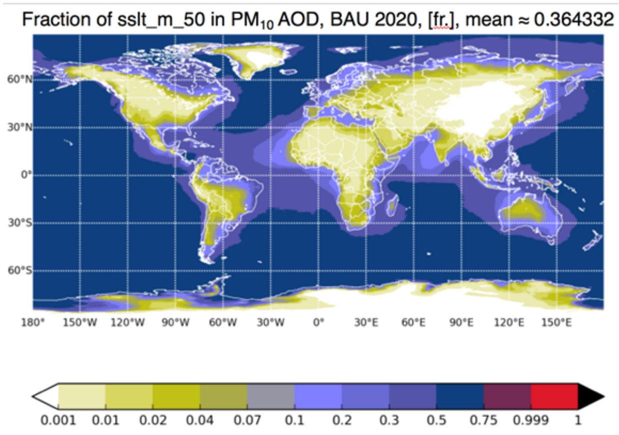

Sea salt, particles 0.1 µm- 1.5 µm dry diameter

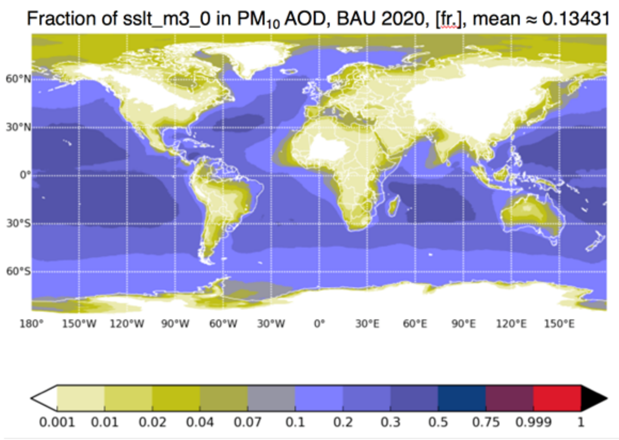

Sea salt, particles 1.5 µm- 6 µm dry diameter

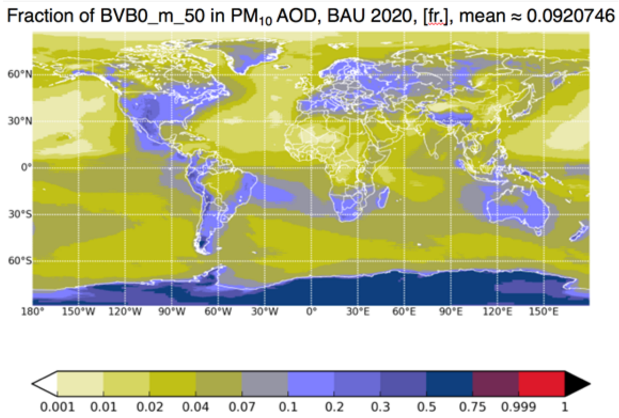

Low-volatility secondary organic particles

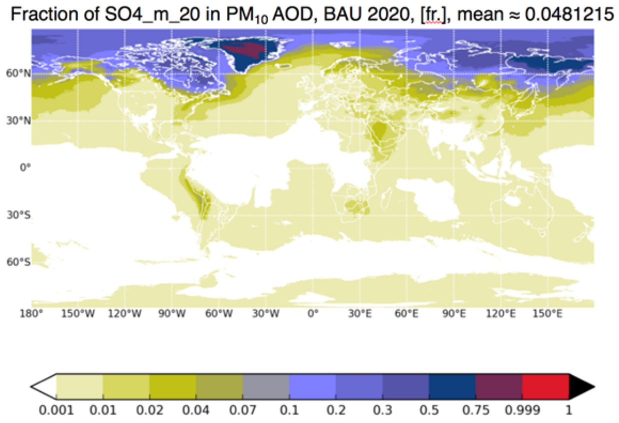

Sulphates 0.1 µm - 0.3 µm dry diameter

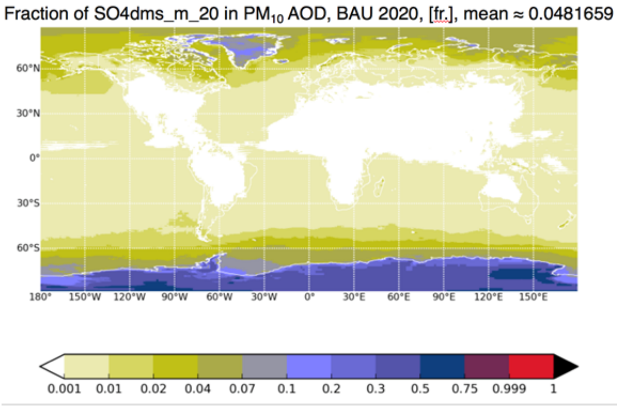

Secondary sulphates from DMS oxidation

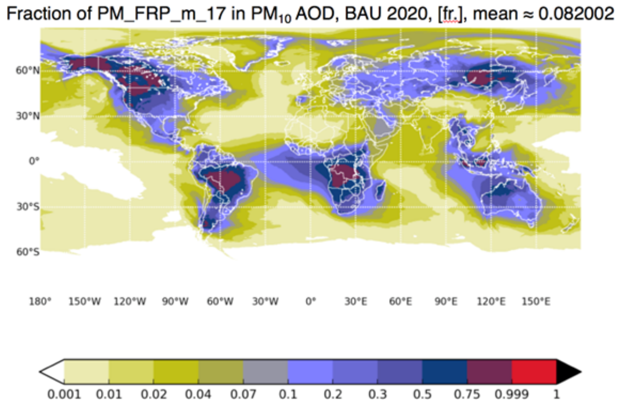

Fire-induced primary PM, 0.01 µm – 0.7 µm dry diameter

Supplementary Figure 8 : Aerosol optical depth contributors

Fractions of the major contributors to total aerosol optical depth (AOD) at 550nm wavelength. (DMS = dimethyl sulphide)

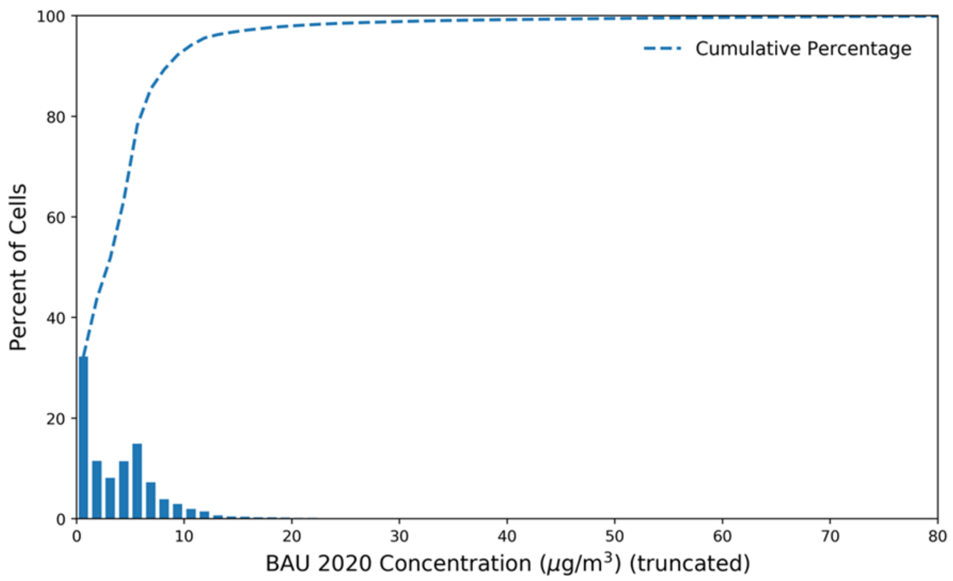

Supplementary Figure 9 : Concentration histogram

Percentage of grid cells by Business-as-usual concentration where population was greater than or equal to 1. Dashed line shows cumulative percentage. These results show that the data are heavily skewed (skewness = 6.13), and the vast majority (99%) of concentration observations are under 34.1 μg m^{-3}.

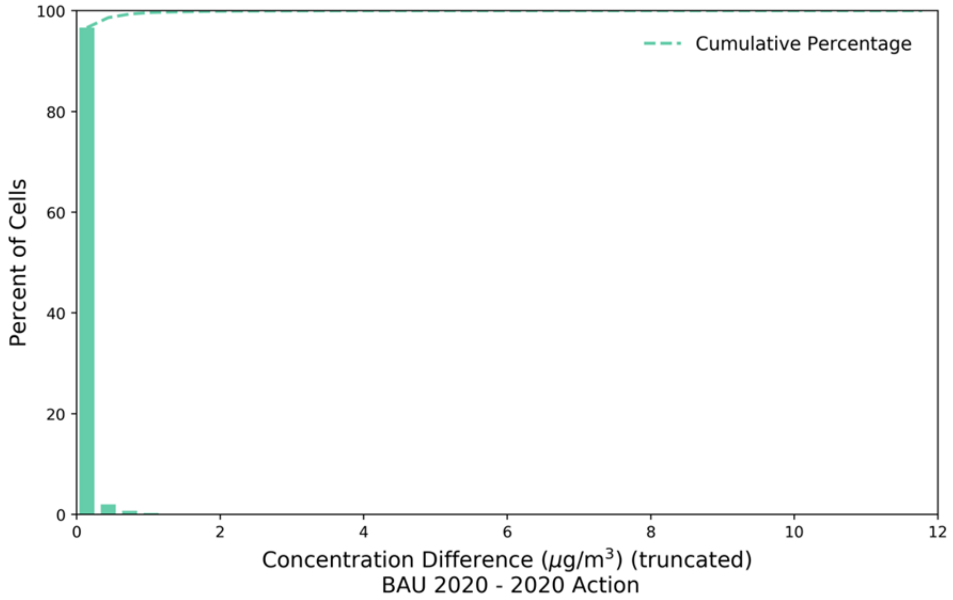

Supplementary Figure 10 : Concentration difference histogram

Percentage of grid cells by concentration difference (Business-as-usual – 2020 Action) where population is greater than or equal to 1. Dashed line shows cumulative percentage. These results show that the data are heavily skewed (skewness = 11.50), and the vast majority (>99%) of concentration differences are under 2 μg m-3.

Supplementary Notes

Supplementary Note 1: Mortality and Morbidity Analysis

Mortality analysis using the Lepeule paramaterization, β coefficients, and health function produce a regional distribution of chronic exposure cardiovascular and lung cancer mortalities. Using the major United Nations regions, Supplementary Table 1 illustrates that most adult mortality aligns with the confluence of intense shipping traffic along trade lanes and dense coastal populations proximal to these trade lanes. However, greater reduction in adult mortality is concentrated in those regions that rely upon the 2020 Action of international fuel-sulphur standards; in Europe, in particular, self-determined similar clean fuels standards implemented independent of the IMO MARPOL VI policy achieve most of the benefits of cleaner fuels.

Estimated childhood asthma morbidity results in regional patterns that differ from the regional distribution of estimated chronic exposure mortality. Supplementary Table 2 presents an asymmetric distribution of health burden from Supplementary Table 1. Supplementary Figure 2 presents the map results for childhood asthma morbidity under both cases, and the geospatial reduction in estimated childhood asthma. Two factors explain this. First, adult population densities and youth population densities vary greatly among nations. Nations with more youth have more cases of childhood asthma for similar exposure to ship-generated particulate pollution. Second, the reported incident rates used in computing asthma morbidity vary among nations. Nations with more advanced health care systems (i.e., typically wealthier nations) report higher underlying incident rates for childhood asthma. The first condition suggests expected differences between mortality and morbidity. The second condition demonstrates one key source of uncertainty in morbidity estimation at global scales discussed in the Methods section.

These childhood asthma estimates likely are conservatively low, because we use annual average concentration differences per grid cell, rather than searching each grid-cell-day and using that cell's given day's maximum difference. Because we perform the calculation one time (not daily), we interpret our results as the number of individual youths who may suffer at least one reportable asthma incidence, i.e., the number of youths with diagnosable asthma wheeze that year. This estimate is comparable, therefore, with the estimated number of persons with asthma that the Global Asthma Report estimates.[7]

Supplementary Note 2: Impact of Geospatial Resolution on Health Results

This analysis uses a geospatial resolution considerably more resolved that other comparable work in the literature. To understand how the geospatial resolution affects results, we compare our 10km x 10km gridded results with results from an analysis done at a 50km x 50km resolution (Supplementary Figure 3). This comparison demonstrates the heterogeneous regional effects that can result due to geospatial resolution. Negative values (red) demonstrate areas where the 50x50 case results in lower concentrations and positive values (blue) demonstrate areas where the 50x50 case results in higher concentrations. Specifically, one can observe that better resolution increases exposure estimates for large parts of Asia, Europe, Western USA port regions, and the Suez Canal region, among other locations.

Supplementary Note 3: Log-linear Risk Functions

Alternative equations using the so-called "log-linear" C-R function and alternative beta coefficients are included for two reasons. First, these equations compare with other global health studies that used

"integrated exposure risk functions," based on log-linear C-R functions from Burnett et al [8]. Second, these equations provide estimates that compare with prior research on global mortality impacts from shipping [9,10].

For our *log-linear* model, relative risk (RR) is defined as:

$$RR = \left[\frac{C_1 + 1}{C_0 + 1}\right]^{\beta}$$

where C_0 and C_1 represent concentration levels for each scenario, and β is derived from empirical 101 studies reported in the literature [11]. Therefore, AF simplifies to:

$$AF = \left\{1 - \left[\frac{C_0 + 1}{C_1 + 1}\right]^{\beta}\right\}$$

which leaves us with the following impact calculation:

$$E = \left\{\left[1 - \left(\frac{1 + C_0}{1 + C_1}\right)^{\beta}\right] BP\right\}$$

Where the log-linear coefficients are β = 0.1551 (95% CI = 0.05624, 0.2541) for cardiopulmonary mortality; β = 0.23218 (95% CI = 0.08563, 0.37873) for lung cancer related mortality. We do not approximate the log-linear β for childhood asthma morbidity because Zheng et al (2015) specify a relative risk for a linear relationship between $PM_{2.5}$ and childhood asthma.[12]

Supplementary Figure 4 shows a comparison of relative risks under linear and log-linear model specifications. The log-linear function produces higher initial RR under low initial concentration (C_0) conditions, but saturates and intersects the linear form at 15 $\mu g\ m^{-3}$. At higher C_0 values, the log-linear function produces lower RR values than the linear model, and appears to imply RR saturation asymptotes (not in evidence from, and potentially inconsistent with, epidemiology of air pollution health impacts.

Supplementary Table 3 presents log-linear C-R function results for ship-related mortality due to cardiovascular disease and lung cancer, and estimated youth population affected by childhood asthma morbidity. Regional distributions using the log-linear C-R functions are presented for mortality estimates in Supplementary Table 4, and for childhood asthma mortality in Supplementary Table 5.

Supplementary Note 4: Analysis of Direct and Indirect Aerosol Effects

We find that the implementation of the IMO global fuel sulfur standard increases the indirect radiative forcing from ships globally from -86 mW m^{-2} (cooling effect) to -19 mW m^{-2} (cooling effect). Thus, the radiative forcing due to the indirect effects from the IMO standard is +67 mW m^{-2} (i.e., the cooling effect is reduced by 67 mW m^{-2}). These indirect effects can be compared to the direct effects that we calculate as -5.2 mW m^{-2} and -1.4 mW m^{-2} for the no action v. action scenarios, respectively (see manuscript Table 3). Combined, the net change in aerosol forcing is calculated at 69 mW m^{-2}, representing a net addition to warming or a loss in aerosol cooling.

At scales of observation for single plumes, ship tracks may have a much smaller equivalent diameter cross-section than the grid size modeled here, even though this grid is more resolved than prior models. A gradient exists between the core of the plume and other points in a modeled gridbox 10x10 km^2. We expect increased negative forcing due to more intensely reflective plumes to increase our results somewhat, but do not attempt to estimate the extent of this potential effect.

A distribution of the effect over the globe (Supplementary Figure 6) demonstrates that the lower sulphur concentrations have significant impact over <5% of the cases (here, one case is 3-hourly mean radiative forcing in 10km $\times$ 10km grid cell). However, the actual effect can exceed 10 Wm^{-2}, albeit very rarely. The fraction of no-impact cases is therefore >95%, half of it being night-time and quarter representing the areas not affect by shipping. Somewhat under 10% of cases are (almost) clear-sky conditions, which may change in the future climate.

Supplementary Note 5: Outline of the SILAM model evaluation

Evaluation of SILAM has been performed in several (inter-)national studies and presented in a series of publications. The most-relevant for the current study are: the comparison over marine areas stressing the performance of the sea salt controlling mechanisms [1-3], analysis of European data [4], publications of Air Quality Model Intercomparison Initiative [5,6], automatic daily evaluation for Europe (http://www.regional.atmosphere.copernicus.eu/), China (http://www.marcopolo-panda.eu/forecast), and Northern Africa and Southern Europe (https://sds-was.aemet.es). Summarizing the outcome of these studies, the mean SO_2 concentrations in the regions with substantial sulphur emission are reproduced very well: in China the bias of SO_2 is -3.3 $\mu g\ S\ m^{-3}$ (mean observed level is 20.1 $\mu g\ S\ m^{-3}$), in Europe, the SO_2 concentration bias is +0.3 $\mu g\ m^{-3}$ (mean observed 4.0 $\mu g\ S\ m^{-3}$) while for sulphate it is -0.03 $\mu g\ S\ m^{-3}$ (observed mean is 0.8 $\mu g\ S\ m^{-3}$). The optical depth over ocean is largely controlled by sea salt, so it is difficult to extrapolate the practically unbiased AOD predictions of SILAM (bias < 10% of the total AOD) to sulphates. Noticeable negative bias is associated only with desert dust plumes, where mean AOD is under-stated by -0.3 : -0.4, owing to uncertain emissions. Nevertheless, the evaluation performed this-far in several institutes and international projects did not show any major deficiencies of the model.

Supplementary Table 1. Linear C-R model central estimates of mortality by UN region, including avoided mortality with 2020 Action.

UNSD Region	BAU Mortality from Ships	Mortality from Ships with 2020 Action	Avoided Mortality with Cleaner Ship Fuels
Asia	316,722	206,896	109,826
Europe	40,162	38,067	2,095
Africa	28,396	11,408	16,988
Latin America and Caribbean	10,862	5,923	7,079
Northern America	6,594	3,783	671
Oceania	488	184	304
**Total**	**403,224**	**266,261**	**136,963**

Supplementary Table 2. Linear C-R model central estimates of morbidity by UN region, including avoided childhood asthma with 2020 Action.

UNSD Region	BAU Asthma from ships	Asthma from Ships with 2020 Action	Avoided Asthma with Cleaner Ship Fuels
Asia	7,237,288	3,145,217	4,092,071
Europe	1,073,691	1,029,679	44,012
Africa	3,770,870	1,285,353	2,485,517
Latin America and Caribbean	1,379,231	471,006	908,225
Northern America	513,785	461,289	52,496
Oceania	68,372	25,541	42,831
**Total**	**14,043,237**	**6,418,085**	**7,625,152**

Supplementary Table 3. Log-linear C-R function results for estimated annual health impacts of global shipping and benefits in 2020 due to implementation of the IMO low sulphur fuel standard.

Scenario Results (Log-Linear C-R Model)	Mortality Estimate (annual premature adult deaths*)		Childhood Asthma (million cases)
**BAU 2020** (No implementation of global 0.5% S fuel standard)	CV† Mortality	87,300 (CI 95% 31,700; 142,400)	**3.1 (CI 95% 1.7; 4.7)**
	LC† Mortality	16,200 (CI 95% 6,000; 26,300)	
	**Combined Mortality**	**103,500 (CI 95% 37,300; 168,700)**	
**2020 Action** (Implementation of global 0.5% S fuel standard in 2020)	CV Mortality	52,500 (CI 95% 19,000; 85,800)	**1.7 (CI 95% 0.8; 2.2)**
	LC Mortality	11,500 (CI 95% 4,300; 18,600)	
	**Combined Mortality**	**64,000 (CI 95% 23,300; 104,400)**	
**Health benefit of 2020 Action**	**Avoided Mortality‡**		**Avoided Morbidity**
	CV: 34800 (CI 95% 12,700; 56,600)		**1.4 (CI 95% 0.9; 2.5)**
	LC: 4700 (CI 95% 1,700; 7,700)		
	**Combined: 39500 (CI 95% 14,400; 64,300)**		

* Values for annual premature mortality are rounded to nearest 100; values for annual childhood asthma morbidity rounded to nearest 100 thousand.
† CV = cardiovascular disease; LC = lung cancer; CI 95% = 95 percent confidence interval.
‡ Differences between avoided health impacts and scenario differences attributed to rounding.

Supplementary Table 4. Log-Linear model central estimates of mortality by UN region, including avoided mortality with 2020 Action.

UNSD Region	BAU Mortality from Ships	Mortality from Ships with 2020 Action	Avoided Mortality with Cleaner Ship Fuels
Asia	57,496	30,568	26,928
Europe	25,592	24,355	6,419
Africa	10,754	4,335	4,161
Latin America and Caribbean	6,039	2,675	1,237
Northern America	3,124	1,878	449
Oceania	450	168	282
**Total**	**103,455**	**63,979**	**39,476**

Supplementary Table 5. Log-Linear C-R model central estimates of morbidity by UN region, including avoided childhood asthma with 2020 Action.

UNSD Region	BAU Asthma from ships	Asthma from Ships with 2020 Action	Avoided Asthma with Cleaner Ship Fuels
Asia	1,178,037	725,565	452,472
Europe	442,095	16,111	425,984
Africa	848,186	557,967	290,219
Latin America and Caribbean	460,671	316,863	143,808
Northern America	146,316	21,271	125,045
Oceania	41,819	26,310	15,509
**Total**	**3,117,124**	**1,664,087**	**1,453,037**

Supplementary References

1 Soares, J. *et al.* Impact of climate change on the production and transport of sea salt aerosol on European seas. *Atmospheric Chemistry and Physics* **16**, 13081-13104, doi:10.5194/acp-16-13081-2016 (2016).

2 Sofiev, M., Soares, J., Prank, M., De Leeuw, G. & Kukkonen, J. A regional-to-global model of emission and transport of sea salt particles in the atmosphere. *Journal of Geophysical Research Atmospheres* **116**, doi:10.1029/2010JD014713 (2011).

3 Tsyro, S. *et al.* Modelling of sea salt concentrations over Europe: Key uncertainties and comparison with observations. *Atmospheric Chemistry and Physics* **11**, 10367-10388, doi:10.5194/acp-11-10367-2011 (2011).

4 Prank, M. *et al.* Evaluation of the performance of four chemical transport models in predicting the aerosol chemical composition in Europe in 2005. *Atmospheric Chemistry and Physics* **16**, 6041-6070 (2016).

5 Solazzo, E. *et al.* Operational model evaluation for particulate matter in Europe and North America in the context of AQMEII. *Atmospheric Environment* **53**, 75-92, doi:10.1016/j.atmosenv.2012.02.045 (2012).

6 Solazzo, E. *et al.* Model evaluation and ensemble modelling of surface-level ozone in Europe and North America in the context of AQMEII. *Atmospheric Environment* **53**, 60-74, doi:10.1016/j.atmosenv.2012.01.003 (2012).

7 Global Asthma Network. *The Global Asthma Report 2014* (2014).

8 Burnett, R. T. *et al.* An integrated risk function for estimating the global burden of disease attributable to ambient fine particulate matter exposure. *Environ Health Perspect* **122**, 397-403, doi:10.1289/ehp.1307049 (2014).

9 Corbett, J., Winebrake, J. & Green, E. An assessment of technologies for reducing regional short-lived climate forcers emitted by ships with implications for Arctic shipping. *Carbon Management* **1**, 207-225, doi:10.4155/cmt.10.27 (2010).

10 Winebrake, J. J., Corbett, J. J., Green, E. H., Lauer, A. & Eyring, V. Mitigating the Health Impacts of Pollution from Oceangoing Shipping: An Assessment of Low-Sulfur Fuel Mandates. *Environmental Science & Technology* **43**, 4776-4782, doi:doi:10.1021/es803224q (2009).

11 Lepeule, J., Laden, F., Dockery, D. & Schwartz, J. Chronic exposure to fine particles and mortality: an extended follow-up of the Harvard Six Cities study from 1974 to 2009. *Environmental health perspectives* **120**, 965 (2012).

12 Zheng, X.-y. *et al.* Association between air pollutants and asthma emergency room visits and hospital admissions in time series studies: a systematic review and meta-analysis. *PLoS One* **10**, e0138146 (2015).
